# Identification of a Muscle-Invasive Bladder Carcinoma Molecular Subtype of Poor Responders to Neoadjuvant Chemotherapy and High Expression of Targetable Biomarkers

**DOI:** 10.3390/ijms27010476

**Published:** 2026-01-02

**Authors:** Lucía Trilla-Fuertes, Jorge Pedregosa-Barbas, Eugenia García-Fernández, Francisco Zambrana, Imanol Martínez-Salas, Pablo Gajate, Fernando Becerril-Gómez, Pedro Lalanda-Delgado, Antje Dittmann, Rocío López-Vacas, Laura Kunz, Gustavo Rubio, Sandra Nieto-Torrero, Ana Pertejo, Pilar González-Peramato, Juan Ángel Fresno Vara, Angelo Gámez-Pozo, Álvaro Pinto-Marín

**Affiliations:** 1 Molecular Oncology Lab, INGEMM, La Paz University Hospital-IdiPAZ, Paseo de la Castellana 261, 28046 Madrid, Spain; lucia.trilla@salud.madrid.org (L.T.-F.); fernando.becerril@salud.madrid.org (F.B.-G.); plalandelg@gmail.com (P.L.-D.); rlvacas@salud.madrid.org (R.L.-V.); juanangel.fresno@salud.madrid.org (J.Á.F.V.); 2 Medical Oncology Service, La Paz University Hospital-IdiPAZ, Paseo de la Castellana 261, 28046 Madrid, Spain; jorge.pedregosa@salud.madrid.org (J.P.-B.); ana.pertejo@salud.madrid.org (A.P.); 3 Department of Pathology, La Paz University Hospital-IdiPAZ, UAM, 28046 Madrid, Spain; euge17@yahoo.com (E.G.-F.); mpilar.gonzalezperamato@salud.madrid.org (P.G.-P.); 4 Medical Oncology Department, Infanta Sofía University Hospital, 28702 Madrid, Spain; francisco.zambrana@salud.madrid.org (F.Z.); gustavo.rubio@slaud.madrid.org (G.R.); 5 Medical Oncology Department, IIS-FJD, University Hospital Fundación Jiménez Díaz, 28040 Madrid, Spain; imanol.martinez@quironsalud.es; 6 Medical Oncology Department, Hospital Universitario Ramón y Cajal, 28034 Madrid, Spain; pgajate@oncologiahrc.com; 7 Functional Genomics Center Zurich, University of Zurich/ETH Zurich, 8057 Zurich, Switzerland; antje.dittmann@fgcz.uzh.ch (A.D.); laura.kunz@fgcz.uzh.ch (L.K.); 8 IdiPAZ Biobank, La Paz University Hospital-IdiPAZ, 28046 Madrid, Spain; biobanco.hulp@salud.madrid.org; 9 Biomedical Research Networking Center on Oncology-CIBERONC, ISCIII, 28029 Madrid, Spain

**Keywords:** muscle-invasive bladder cancer, proteomics subtype, response to neoadjuvant chemotherapy, targetable biomarkers

## Abstract

Neoadjuvant chemotherapy (NACT) is the standard treatment for muscle-invasive bladder carcinoma (MIBC), but its efficacy varies significantly among patients. The aim of this study is the identification of biomarkers and biological processes related to the response to neoadjuvant chemotherapy (NACT) in muscle-invasive bladder carcinoma (MIBC). Fifty-eight transurethral resection (TURBT) samples and thirty cystectomy samples from NACT non-responders were analyzed using mass spectrometry. Samples were classified with sparse k-means and consensus clustering. Protein networks were built using probabilistic graphical models, grouped into functional nodes, and analyzed for activity differences. Gene set enrichment analysis was applied to identify resistance mechanisms, and results were validated using The Cancer Genome Atlas (TCGA) cohort. Proteomic analysis revealed two independent classifications in TURBT samples. The first (Layer1) divided tumors into three groups, including one NACT non-responder subtype not aligned with traditional luminal or basal classifications but characterized by high expression of targetable markers NECTIN4 and Her2. The second (Layer3) separated luminal-papillary tumors from luminal-infiltrated/luminal and basal tumors. While Layer3 groups did not differ in NACT response, they showed distinct disease-free survival outcomes. Importantly, complete response to NACT was linked to improved survival in luminal subgroups but not in basal tumors, suggesting subtype-specific prognostic implications. Finally, analysis of cystectomy samples identified unique mechanisms of resistance for each subgroup, suggesting tailored therapeutic approaches. Two classification systems were defined as follows: one identified a proteomics-based non-responder group with actionable targets, and the other linked tumor subtype to prognosis. Distinct resistance mechanisms suggest opportunities for subtype-specific therapies, supporting improved management and treatment development for MIBC patients.

## 1. Introduction

Bladder carcinoma is the eighth tumor in incidence worldwide with 614,298 new cases and 220,596 deaths in 2022 [1]. In localized muscle-invasive bladder carcinoma (MIBC), the standard treatment is neoadjuvant chemotherapy (NACT) based on cisplatin followed by cystectomy. The benefit showed by NACT in overall survival (OS) is 8% at 5 years, especially focused on the 30–40% of the patients that have achieved a pathological complete response. However, the selection in clinical practice for receiving NACT is based on patient health status and renal function [2].

Recently, it has been published in a phase III clinical trial that the adjuvant administration of nivolumab improves disease-free survival (DFS) in PDL-1 positive patients not eligible for receiving NACT or those that not achieve a complete response to NACT [3]. Also, the combination of Durvalumab with neoadjuvant cisplatin-based chemotherapy, together with adjuvant Durvalumab following cystectomy, also improves survival outcomes [4].

MIBC is a molecularly heterogeneous disease with different responses to treatment and evolution. Therefore, it is necessary that an in-depth molecular characterization and the identification of biomarkers be capable of predicting a response to NACT.

Molecular classifications of MIBC based on transcriptomics data have been previously established. Choi et al. identified the following three MIBC transcriptomics subtypes: basal, luminal, and p53-like, which expressed both luminal biomarkers and an activated wild-type p53 expression. These p53 tumors had a lower response rate to NACT [5]. The Cancer Genome Atlas (TCGA) extended this classification to the following five groups: luminal-papillary, luminal-infiltrated, luminal, basal/squamous, and neuronal subtypes [6]. A re-analysis of MIBC TCGA data performed by our group suggested the following two independent classifications: one based on immune features and a luminal/basal classification [7]. Finally, a consensus molecular classification of MIBC was published in 2020, refining the TCGA classification by adding a new luminal subtype (luminal unstable) [8].

The association between MIBC molecular subtypes and response to NACT is controversial. There are studies suggesting no association between molecular subtypes and response to NACT [9], while others postulate that basal tumors have a better response to NACT [10].

Proteomics studies of MIBC are less frequent than transcriptomics studies. Our group identified two proteomics subtypes in muscle-invasive urothelial carcinoma, one characterized by higher metabolism and adhesion activity and the other by a higher immune response activity [11]. Groeneveld et al. analyzed non-muscle invasive and MIBC samples and identified five proteomics subgroups that partially overlapped with transcriptomics subtypes [12]. Dressler et al. analyzed one hundred and ninety-six patients from all urothelial carcinoma stages, including non-muscle invasive and MIBC samples, identifying also five proteomics clusters [13].

The aim of this study is using high-throughput proteomics to find molecular characteristics related to response to NACT, analyzing both transurethral resection of bladder tumor (TURBT) and cystectomy samples from MIBC patients.

## 2. Results

### 2.1. Clinical Characteristics of the EPIC-MIBC Cohort

Fifty-eighth MIBC patients with available treatment-naïve TURBT FFPE sample were included in this study. Twenty-four patients achieved a pathological complete response (CR) to NACT while thirty-two patients did not fully respond to NACT. Of those patients, thirty cystectomies (after NACT) were also analyzed. The two remaining cystectomies could not be collected. Finally, response to NACT was unknown for two patients. Fourteen patients suffered a relapse (three that achieved a CR and ten that did not fully respond to NACT) and eleven died after a median of follow-up of 30 months after surgery. On the contrary, twenty-one of the patients that achieved a CR and twenty-two of the non-responder patients did not relapse. In both cases, the median of cisplatin-based NACT cycles received by responder and non-responder patients was three. Detailed clinical information can be found in Table 1.

### 2.2. Proteomics Experiments

EPIC-MIBC samples, both TURBT and cystectomy, were analyzed using DIA MS proteomics. In these experiments, 5532 proteins were identified and quantified. After applying quality criteria, 3378 of these proteins were used for subsequent analyses.

### 2.3. Classification of EPIC-MIBC TURBT Samples

#### 2.3.1. Protein Network Analysis in EPIC-MIBC TURBT Samples

A network of protein relations, based on the obtained proteomics data in TURBT samples, was built using PGMs. The network was split in 52 nodes (groups of proteins), according to the Louvain community algorithm, and the overrepresented biological function for each node was assessed (Supplementary Figure S1, Supplementary Table S1). To study the differential biological processes between groups of samples, functional node activities were calculated as a representative measure for each functional node. A detailed composition of each functional node can be found in Supplementary Table S1.

#### 2.3.2. Layer1 Classification: DNA Replication and Adhesion

First, we explored the different layers of biological information applying recursively sparse k-mean and CC. The first classification obtained by this method, Layer1, is based on 85 proteins and shows enrichment in DNA replication and focal adhesion proteins. Interestingly, the list of 85 proteins that defines this layer includes NECTIN4 and ERBB2 (Her2) (Supplementary Table S2). This layer classified MIBC TURBT samples into three groups (Layer1.1 = 16 [27.5%] patients, Layer1.2 = 9 [15.5%] patients, and Layer1.3 = 33 [57%] samples).

The protein network and functional node activities were used to characterize the differential biological processes between Layer1 groups. From a functional point of view, Layer1.2 presented higher activity of immune response and actin cytoskeleton and extracellular matrix functional nodes, coupled with lower expression of mRNA processing and spliceosome, proliferation, defense immune response, ribosome and translation, and mitochondrial metabolism functional nodes when compared with Layer1.1 and Layer1.3 tumors. Additionally, Layer1.1 and Layer1.3 groups showed some differences as follows: mRNA processing and spliceosome nodes, endoplasmic reticulum transport, and telomerase and microtubules are higher in Layer1.1, whereas cytoskeleton and extracellular matrix, immune response and antigen binding, and cellular detoxification nodes presented higher functional activity in Layer1.3 tumors (Supplementary Figure S2).

Regarding the expression of luminal/basal biomarkers, Layer1.2 presented higher expression of luminal-infiltrated biomarkers such as DES, PGM5, and SGCD (Supplementary Figure S3), whereas no specific markers were found for Layer1.1 and Layer1.3 groups. Finally, we studied the relation between Layer1 groups and the MIBC consensus molecular classification previously described. Layer1.2 included NE-like and stroma-rich tumors, whereas Layer1.1 and 1.3 included tumors from all consensus groups (Supplementary Figure S4). Therefore, this Layer 1 classification is independent of luminal and basal subtypes.

**Figure 1:**
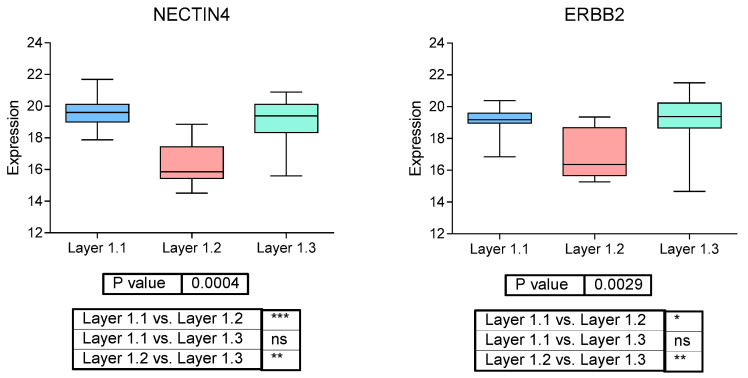
Protein expression of NECTIN4 and ERBB2 (Her2) in EPIC-MIBC TURBT samples according to Layer1 groups. ns *p* > 0.05; * *p* ≤ 0.05; ** *p* ≤ 0.01; *** *p* ≤ 0.001.

As mentioned above, the list of proteins defined by classified samples into Layer1 includes NECTIN4 and ERBB2 (Her2), both targetable proteins in MIBC. Therefore, we studied their expression in each Layer1 group. Indeed, Layer1.1 and Layer1.3 (NACT non-responders) had a significantly higher NECTIN4 and ERBB2 protein expression than Layer1.2 (Figure 1).

#### 2.3.3. Layer2 Classification: Cytoskeleton

After applying sparse k-means and CC to the remaining proteins, a new classification, Layer2, was obtained. It was based on 107 proteins, mostly related to cytoskeleton (n = 13). This Layer2 classification is equivalent to Layer1 (Supplementary Figure S5).

#### 2.3.4. Layer3 Classification: Innate Immune Response

Layer3 classification was based on 80 proteins, with an enrichment of innate immune response-related proteins (Supplementary Table S3). It divided the samples into two groups as follows: Layer3.1 (n = 29 [50%] patients) and Layer3.2 (n = 29 [50%] patients).

Regarding the functional characterization of these two groups, Layer3.1 tumors presented higher proliferation, regulation of nucleotide-excision repair, cell migration, and DNA repair, mitochondria and mRNA processing nodes, whereas Layer3.2 group showed higher immune functional node activities (Supplementary Figure S6).

**Figure 2:**
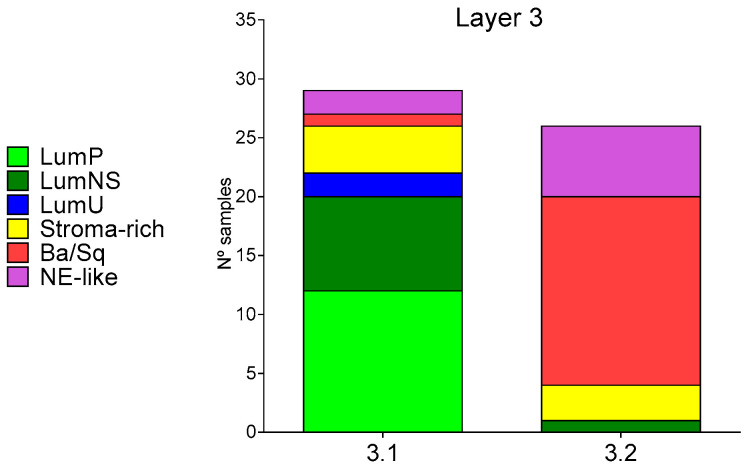
Distribution of MIBC consensus molecular subtypes in Layer3 groups of EPIC-MIBC TURBT samples.

Regarding the expression of luminal and basal biomarkers, Layer3.1 tumor presented higher expression of KRT20, a luminal-papillary biomarker, whereas Layer3.2 had higher expression of KRT5, KRT6a, KRT14, and CD44 basal biomarkers, but also PGM5, DES, and SGCD luminal-infiltrated biomarkers (Supplementary Figure S7). Finally, we studied the relation between Layer3 groups and the MIBC consensus molecular classification previously described. Layer3.1 includes most LumP, LumNS, and LumU tumors, whereas most Ba/Sq tumors are included in Layer3.2. NE-like and stroma-rich tumors are split between both groups (Figure 2).

#### 2.3.5. Relation Between Layers, Response to NACT and DFS

**Figure 3:**
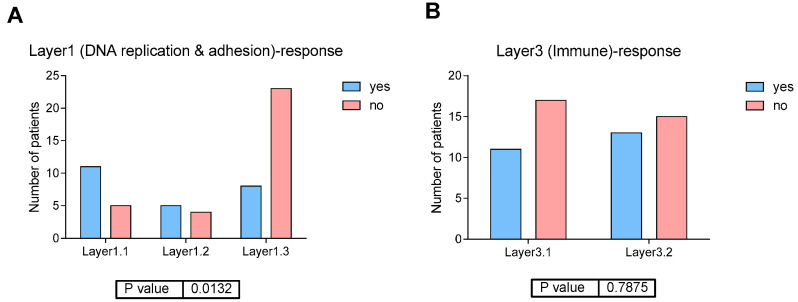
(**A**) Distribution of response to NACT in Layer1 groups in EPIC-MIBC TURBT samples. (**B**) Distribution of response to NACT in Layer3 groups in EPIC-MIBC TURBT samples.

**Figure 4:**
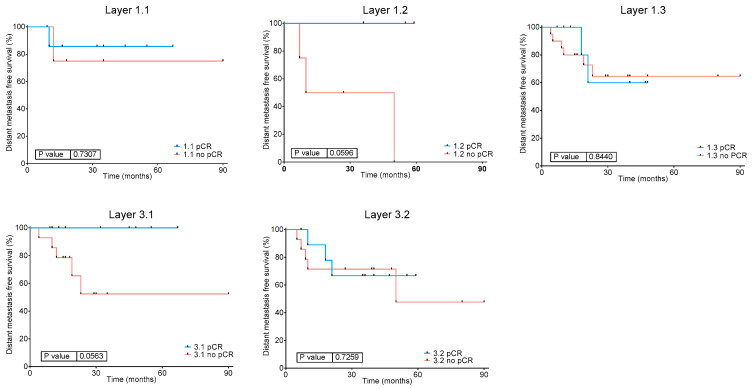
Disease-free survival in Layer1 and Layer3 groups according to response to NACT. pCR: complete response.

Interestingly, Layer1 groups showed a differential distribution of NACT response, with Layer1.3 group mostly formed by NACT non-responders (*p* = 0.013) (Figure 3A), although this does not translate to significant differences in DFS (Supplementary Figure S7). On the other hand, Layer3 classification was not related to response to NACT, being the percentage of responders in Layer3.1 of 39% and in Layer3.2 of 46% (*p* = 0.78) (Figure 3B). Although no significant differences in DFS were found between the Layer3 groups, a trend of a better prognosis in Layer3.1 (formed by luminal tumors) has been observed (Supplementary Figure S8). Finally, we explored the relation between layer classification, response to NACT and DFS. Interestingly, patients with Layer1.2 (NE-like and stroma-rich tumors) and Layer3.1 (luminal) tumors showed a significant relationship between CR and DFS, which is not observed for the other groups. These results suggest that CR is related to a favorable DFS in patients with luminal, NE-like, and stroma-rich tumors, but not in patients with Ba/Sq tumors (Figure 4).

We found differences in five functional node activities between tumors from Layer3.1 (luminal) regarding its response, three related with cytoskeleton and extracellular matrix, one related with cellular detoxification, and one related with immune response (Supplementary Figure S9). No differences in node activity were found in Layer3.2 tumors regarding its response to NACT, probably because of the reduced number of samples in this group.

### 2.4. Validation of the Layer Classification in BLCA-TCGA Cohort

In order to validate our layer classifications, muscle-invasive bladder carcinoma TCGA (BLCA-TCGA) cohort was used. After classifying BLCA-TCGA cohort in each layer group, functional node activities from differential biological processes between layer groups were checked. It was confirmed that Layer1.2 in TCGA cohort also had higher immune response, cytoskeleton, adhesion and extracellular matrix, and cellular detoxification, whereas Layer1.3 presented higher endoplasmic reticulum activity and Layer1.1 had higher telomerase and microtubules node activity (Supplementary Figure S10). Regarding Layer3, it was confirmed that Layer3.1 had higher mRNA processing and spliceosome, mitochondrial metabolism and translation, regulation of cell cycle, cell–cell adhesion and migration, and DNA repair activities, while Layer3.2 showed higher immune response and focal adhesion and cytoskeleton (Supplementary Figure S11).

Unfortunately, it was not possible to check differences in response to NACT between groups due to most of the BLCA-TCGA patients having been treated with adjuvant schemes.

**Figure 5:**
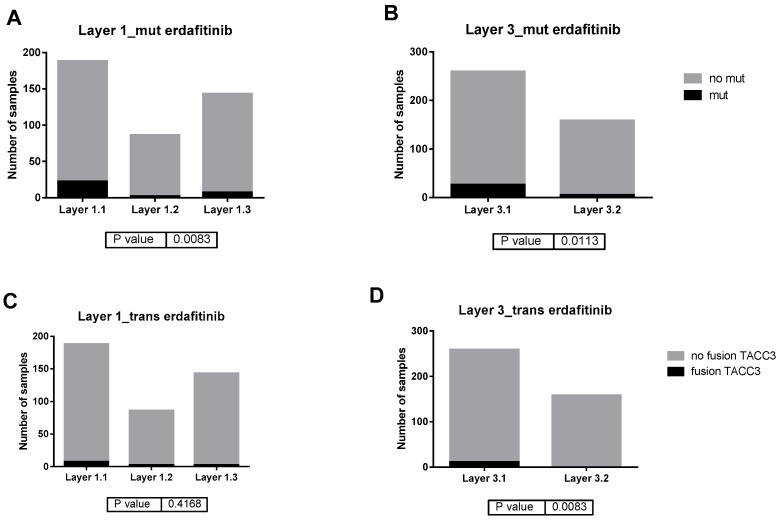
FGFR3 alterations in BLCA-TCGA cohort. (**A**) Distribution of R248C, S249C, G370C, and Y373C FGFR3 mutations in Layer1 groups. (**B**) Distribution of R248C, S249C, G370C, and Y373C, FGFR3 mutations in Layer3 groups. (**C**) Distribution of FGFR3-TACC3 fusions in Layer1 groups. (**D**) Distribution of FGFR3-TACC3 fusions in Layer3 groups.

Interestingly, BLCA-TCGA cohort has available data about FGFR3 genetic alterations. Therefore, we studied the distribution of FGFR3 alterations defined as targetable by erdafitinib according to its technical sheet, including R248C, S249C, G370C, and Y373C punctual mutations, and fusions with TACC3 gene. Interestingly, FGFR3 alterations distribution is different between layer classifications, being more frequent in Layer1.1 and Layer3.1 (Figure 5).

### 2.5. Search for Mechanisms of Resistance to NACT in EPIC-MIBC Cohort Analyzing Cystectomy Samples

Finally, with the aim of identifying mechanisms of resistance related to cancer hallmarks, cystectomy samples (after NACT treatment) from non-responder patients in EPIC-MIBC cohort were collected.

Layer1.1 cystectomies were enriched in DNA repair, genes upregulated by KRAS activation, p53 pathway, apical surface, Wnt beta catenin signaling, E2F targets, inflammatory response, G2M checkpoint, estrogen response early, interferon alpha response, and Myc targets (FDR < 5%).

Layer1.2 cystectomy samples were enriched in angiogenesis (FDR < 5%).

Layer1.3 cystectomies had an enrichment in myogenesis, epithelial–mesenchymal transition, coagulation, heme metabolism, and genes downregulated by KRAS activation (FDR < 5%).

In Layer3.1 group no enriched processes were identified, while myogenesis was enriched in Layer3.2 (FDR < 5%).

**Figure 6:**
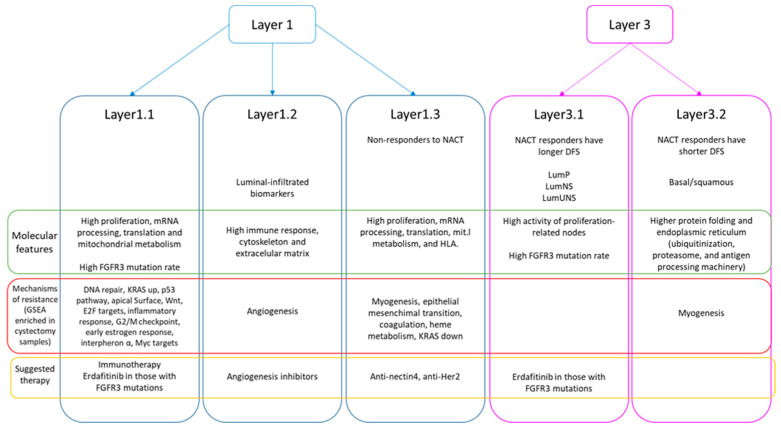
Summary of the findings of this study.

A summary of the findings of this study can be found in Figure 6.

## 3. Discussion

Neoadjuvant cisplatin-based chemotherapy is the standard of care in MIBC. However, its benefit is only an 8% and response to NACT in patients diagnosed with MIBC, which presented similar clinical characteristics, is heterogeneous [2]. Several efforts have been made to relate MIBC transcriptomic subtypes with response to NACT, but results are controversial [5,10].

In this work, we used high-throughput proteomics to characterize both TURBT and cystectomy samples from MIBC patients that received NACT in order to deeply characterize the mechanisms involved in response and resistance to this treatment.

First, TURBT pre-treatment samples were analyzed in order to find molecular features related to response to NACT. To classify TURBT samples into molecular subgroups, a layer analysis based on sparse k-means and CC was used. This analysis has demonstrated its utility in previous works at the time to identify different tumor realities (molecular features, immune features, processes related to metastasis, etc.) [7,14,15]. In this case, this approach identifies two independent classifications, which we named Layer1 and Layer3.

Layer1 identified three different groups, with a differential response rate to NACT being Layer1.3, mostly formed by tumors from non-responders to NACT patients. Layer1.2 expressed luminal-infiltrated biomarkers and included only NE-like and stroma-rich tumors. Layer1.1 and Layer1.3 groups did not show a relationship with neither luminal or basal biomarkers nor with consensus molecular subtypes, hence this classification reflects molecular characteristics not related to luminal and basal features.

Layer1.2, consistently with the overexpression of luminal-infiltrated biomarkers, also had a higher activity of the immune response nodes but also of extracellular matrix and actin cytoskeleton. An overexpression of extracellular matrix in luminal-infiltrated and luminal tumors has been reported by TCGA classification [16]. Layer1.1 and Layer1.3 shared some characteristics, presenting a higher activity of mRNA processing and spliceosome, proliferation, defense immune response, ribosome and translation, and mitochondrial metabolism. Metabolic diversity in MIBC has been established by using MIBC cell lines, presenting evidence that OXPHOS and mitochondrial metabolism can contribute to some MIBCs but not to others [17].

Layer1.1 and Layer1.3 also differ in some functional processes that may be the cause of most non-responder patients in the Layer1.3 group but not in Layer1.1. Layer1.1 had some higher mRNA processing and spliceosome nodes, endoplasmic reticulum transport, and telomerase and microtubules functional node activities. On the contrary, Layer1.3 group had higher cytoskeleton and extracellular matrix, immune response and antigen binding, and cellular detoxification nodes. Immune response and antigen binding node contained HLA proteins, belonging to major histocompatibility complex I and II. HLA proteins of complex II are only expressed in antigen presentation cells [18]. A previous work established that a higher expression of HLA-DRA is associated with progression of non-muscle invasive bladder carcinoma to MIBC [19]. Nodes of extracellular matrix and cytoskeleton with higher activity in Layer1.3 group contained collagens, laminins, tubulin, integrins, and filamins. Robertson et al. also identified a cluster in their proteomics data, obtained by reverse protein phase microarrays, with an elevated expression of extracellular matrix proteins (including fibronectin and collagens) that presented a worse survival outcome [6]. However, TCGA MIBC cohort is mostly formed by MIBC tumors treated with adjuvant chemotherapy, which is currently not the standard of care. Finally, the node of cellular detoxification contained proteins involved in cellular oxidoreductase activity, including superoxide dismutase 1 and enzymes involved in glutathione metabolism. Interestingly, the role of glutathione metabolism in response to NACT in MIBC has been previously suggested, establishing that glutathione metabolism genes are enriched in NACT MIBC resistance tumors. In this work, the authors also pointed out that NACT-resistant tumors were not consistent with the published MIBC transcriptomics molecular subtypes [20]. Therefore, it seems that Layer1.3 present a series of molecular features that causes a worse response to NACT, not related to previous transcriptomics-based molecular classifications.

Among the proteins used to establish this classification were NECTIN4 and ERBB2, also known as Her2. Indeed, Layer1.3 had higher expression of NECTIN4 and ERBB2. NECTIN4 expression, measured by immunohistochemistry, is heterogeneous among urothelial subtypes, being more frequent in luminal tumors [21]. Enfortumab-vedotin, an anti-NECTIN4 antibody-drug conjugate (ADC), plus pembrolizumab, has been recently approved for the treatment of metastatic urothelial carcinoma [22] due to its impact in PFS and OS shown in phase III clinical trial [23]. NECTIN4 expression positively correlates with response to enfortumab-vedotin [24]. In addition, trastuzumab-deruxtecan is a Her2 ADC combined with a topoisomerase inhibitor payload that has also been tested for bladder cancer [25]. DESTINY-PanTumor02 clinical trial, which tested trastuzumab-deruxtecan in Her2+ locally advanced or metastatic solid tumors, includes 41 Her2+ bladder tumors that showed a 56% of objective response rate (ORR) in Her2 IHC3+ and 39% of ORR in all bladder cancer patients [26]. Therefore, these two treatments may be considered in these Layer1.3 patients that do not respond to NACT.

The Layer3 classification identified two groups related with basal/luminal characteristics. Layer3.1 include luminal-papillary tumors while Layer3.2 was composed by basal and luminal-infiltrated/luminal tumors and Layer3.1 had a better DFS than Layer3.2, although *p*-value is not significant. A better OS for luminal-papillary tumors compared to the other luminal subtypes has been previously established by TCGA [6].

These two groups did not present a different rate of response to NACT. However, Layer3.1 (luminal) patients showed a significant relationship between CR to NACT and DFS. The relationship between CR and DFS in NACT-treated MIBC has been previously established, and achieving a CR to NACT is considered a good prognosis factor [27]. However, it seems that other factors have a role in this good prognosis. With the aim of identifying these other factors involved in the good prognosis of achieving a CR, differences between Layer3.1 according to response to NACT were studied. Non-responder tumors from Layer3.1 had higher activity of nodes related with cytoskeleton, extracellular matrix, and immune response, specifically complement proteins. Extracellular matrix and cytoskeleton functional nodes are mostly composed by collagens, laminins, tubulin, integrins, and filamins. In the TCGA study, Robertson et al. identified a group with high expression of extracellular matrix proteins, including fibronectin and collagens, that presented a worse survival outcome [6].

These two classifications and the differential biological processes between the defined groups have been validated using the BLCA-TCGA cohort. Unfortunately, response to NACT cannot be studied in this cohort due to the fact that most of the samples were treated by adjuvant schemes. Using WES and data of fusion events available from BLCA-TCGA cohort, we also studied the distribution of FGFR3 alterations targetable by erdafitinib in the defined groups. Erdafitinib is a pan-FGFR inhibitor approved for treatment of unresectable or metastatic urothelial carcinoma [28,29]. FGFR3 mutations and gene fusions are infrequent in Layer1.1 and Layer3.2 (basal) groups, and more frequent in Layer1.1 and Layer3.1 (luminal) tumors. This is not surprising, as FGFR3 alterations are more frequent in luminal tumors, especially in luminal-papillary [5,6].

Finally, with the aim of identifying possible mechanisms of resistance to NACT, GSEA was conducted in cystectomy post-treatment samples from non-responder patients in EPIC-MIBC cohort. GSEA showed differences in the possible mechanisms of resistance between the groups defined in Layer1. Layer1.1 cystectomies were enriched in DNA repair, genes upregulated by KRAS activation, p53 pathway, apical surface, Wnt beta catenin signaling, E2F targets, inflammatory response, G2M checkpoint, estrogen response early, interferon alpha response, and Myc targets. Choi et al. study suggested p53 as involved in resistance to NACT and hypothesized that all chemoresistant tumors adopt a p53 phenotype after receiving chemotherapy [5]. In addition, defects in DNA repair genes predicted response to NACT in MIBC [30]. Moreover, interferon alpha and an inflammatory profile are predictive factors of a good response to immunotherapy [31,32]. Recently, nivolumab has demonstrated its efficacy in improving DFS in MIBC patients that were not eligible for NACT or had not achieved a complete response and also had a positive PDL-1 expression [3]. Therefore, this group may have a good response to immunotherapy after receiving NACT and cystectomy.

Layer1.2 cystectomy samples were enriched in angiogenesis, which plays a key role in bladder cancer progression [33,34]. However, treatment with anti-angiogenic molecules showed modest results in advanced urothelial carcinoma [35]. Maybe a selected population approach could improve benefit of anti-angiogenic treatments.

Layer1.3 cystectomies had an enrichment in myogenesis, epithelial–mesenchymal transition, coagulation, heme metabolism, and genes downregulated by KRAS activation (FDR < 5%). Therefore, the non-responder cystectomies of this group are enriched in processes directly involved in metastasis and cellular migration.

The previous efforts to characterize mechanisms of resistance were done in the whole cohort of MIBC patients but, seeing these results, it may be more informative to perform these analyses in each MIBC group or subtype. Moreover, the mechanisms of resistance proposed here could guide the subsequent treatments in those patients that do not respond to NACT.

Our study has some limitations. First of all, this is a retrospective study with a limited number of samples. Although some of the findings were validated in BLCA-TCGA cohort, a validation in a large retrospective or in a prospective cohort may be interesting. Additionally, in vitro and in vivo validations of the role of Nectin4 and Her2 regarding treatment response would be necessary. The performed proteomics is a non-directed technique, so we did not know the status of all TCGA markers to define Robertson’s molecular subtypes. In addition, we did not know the FGFR3 status of the tumors in the EPIC-MIBC cohort, a point that is gaining interest with the possible use in the future of FGFR3 inhibitors. Also, validating these findings in a cohort that includes patients treated with immunotherapy after NACT and cystectomy could help confirm the possible benefit of immunotherapy in this group of patients.

## 4. Materials and Methods

### 4.1. EPIC-MIBC Cohort

MIBC patients were recruited from four different hospitals (La Paz University Hospital, Ramón y Cajal University Hospital, Jiménez Díaz Foundation, and Infanta Sofía University Hospital). The inclusion criteria were patients over 18 years old, diagnosed with MIBC stages II-IV through TURBT, having received a cisplatin-based NACT followed by cystectomy, with available FFPE TURBT sample. The exclusion criterion was tumors with more than 50% of the tumor with other histology than urothelial (neuroendocrine, squamous, etc.). Pathological response to NACT was determined in the cystectomy sample. Approval from La Paz University Hospital Ethical Committee was obtained (PI-4885) and written informed consent was obtained for each participant in the study. TURBT and cystectomy (when available) formalin-fixed paraffin embedded (FFPE) samples were retrieved for each patient.

### 4.2. Protein Isolation

Proteins were isolated from FFPE samples using a previously described protocol [36,37]. Briefly, FFPE sections were deparaffinized in xylene and washed twice in ethanol. Proteins were extracted using a protocol based on heat antigen retrieval and prepared in 2% SDS. Then, protein isolates were digested with trypsin (1:50), and SDS was removed using Detergent Removal Spin Columns (Pierce). The peptide concentration was determined using a Lunatic UV/Vis polychromatic spectrophotometer (Unchained Labs, Pleasanton, CA, USA). All samples were diluted to 0.04 absorbance.

### 4.3. Liquid Chromatography-Mass Spectrometry Analysis

Mass spectrometry analysis was performed on an Orbitrap Fusion mass spectrometer (Thermo Scientific, Waltham, MA, USA) equipped with a Digital PicoView source (New Objective, Littleton, CO, USA) and coupled to an M-Class UPLC (Waters, Milford, CT, USA). Solvent composition at the two channels was 0.1% formic acid in water for channel A and 0.1% formic acid in acetonitrile for channel B. For each diluted sample 2 µL of peptides were loaded on a commercial MZ Symmetry C18 Trap Column (100 Å, 5 µm, 180 µm × 20 mm, Waters) followed by nanoEase MZ C18 HSS T3 Column (100 Å, 1.8 µm, 75 µm × 250 mm, Waters). The peptides were eluted at a flow rate of 300 nL/min. After a 3 min initial hold at 5% B, a gradient from 5 to 22% B in 80 min and 22 to 32% B in additional 10 min was applied. The column was cleaned after the run by increasing to 95% B and holding 95% B for 10 min prior to re-establishing loading condition for another 10 min. Samples were acquired in a randomized order. The mass spectrometer was operated in data-independent mode (DIA), acquiring a full-scan MS (350−2000 *m*/*z*) at a resolution of 120,000 at 200 *m*/*z*. Followed by HCD (higher-energy collision dissociation) fragmentation on 35 windows with 20 *m*/*z* width between 400 and 1100 *m*/*z*. The MS2 resolution was set to 30,000 and a normalized collision energy of 30% was used. The samples were acquired using internal lock mass calibration on *m*/*z* 371.1012 and 445.1200.

The mass spectrometry proteomics data were handled using the local laboratory information management system (LIMS) [38].

### 4.4. Spectral Library Generation and Protein Quantification

The identification and quantification of proteins from MS data was performed using the DIA-NN workflow (version 1.8.2) [39]. The following parameters for the library-free search were used: precursor charge +2, +3 and +4, precursor mass range 300 *m*/*z* to 1500 *m*/*z*, fragment mass range 200 *m*/*z* to 1800 *m*/*z*, mass accuracy MS1 15 ppm, MS2 15 ppm, enzyme specificity trypsin/P allowing one missed cleavage, and fixed modification carbamidomethylation of cysteine and as variable modification the oxidation of methionin. The in silico spectra were generated using a canonical protein database for humans, concatenated with common protein contaminants (11 August 2023-reviewed-contam-UP000005640.fasta), and the maximum false discovery rate (FDR) was set to 0.01.

### 4.5. Proteomics Data Pre-Processing

Proteomics data was log2-transformed. A quality criterion of at least 75% of valid values in each protein in at least one group (TURBT or cystectomy samples) was applied and missing values were imputed to a normal distribution. These steps were performed using Perseus software [40]. A possible batch effect due to the different origin of the samples was corrected using limma R package v3.46.0 [41].

### 4.6. Biological Layer Analysis

Sparse k-means and consensus cluster (CC) were used to identify independent classifications in the EPIC-MIBC proteomics data, as shown in previous works [7]. First, sparse k-means were used to rank the proteins according to their relevance in the sample classification. Then, CC was used to classify the samples and identify the optimum number of groups using the proteins selected by the sparse k-means. After that, these proteins were removed from the dataset and this workflow was performed again to identify another independent layer based on another molecular characteristic. Sparse k-means were calculated using sparcl R package v1.0.3 [42] and CC was performed using ConsensusClusterPlus R package v.1.68.0 [43]. Gene ontology analyses were performed for each protein list to identify the relevant processes in which they are involved using DAVID webtool [44].

### 4.7. Systems Biology Analyses

Probabilistic graphical models (PGMs) compatible with high-dimensional data were applied to the obtained proteomics data with the aim of building a protein relationship network. The graphs were built in two steps as follows: first, the spanning tree that maximizes likelihood was established, and then, a forward addition of edges that minimizes the Bayesian Information Criterion (BIC) but preserves graph’s decomposability was performed [45]. The PGMs were built using functions based on grapHD R package v.0.2.2 [46] implemented in Python. The obtained network was sought for functional structure, establishing functional nodes in the network with an overrepresented biological function using the Louvain community detection algorithm [47]. The overrepresented biological function was determined using the GOAtools Python package version 3.x and DAVID knowledge based on Gene Ontology (GO) [44]. GO categories enriched in the complete dataset were identified and excluded from the analysis. Then, GO categories significantly overrepresented in each functional node were defined, using an FDR adjusted *p*-value < 0.05 on Fisher’s exact test. For comparisons between groups of samples, functional node activities were calculated as in previous studies [36]. Briefly, the functional node activity is the mean expression of those proteins in each node. Gene Set Enrichment Analysis (GSEA) was performed using GSEA software and hallmarks database as background [48]. Processes were considered as significantly enriched when FDR < 5%.

### 4.8. Consensus Molecular Classification Sample Assignment

Samples were assigned to the consensus molecular classification subtypes using the centroids provided by Ref. [8]. Pearson coefficients were calculated for each sample and each centroid, and a sample was assigned to the consensus molecular subtype (CMS) with the highest Pearson correlation coefficient.

### 4.9. Validation of Layer Classifications in BLCA-TCGA Cohort

RNA-seq data from muscle-invasive bladder cancer TCGA cohort (BLCA-TCGA) was used to validate the replicability of the layer classifications defined in this study. RNA-seq data were obtained from firebrowse.org. Genes defined by the sparse k-means for each classification were used to classify BLCA-TCGA cohort in our layers applying a CC. Then, differential biological processes between our groups were checked in BLCA-TCGA cohort calculating functional node activities.

### 4.10. Study of Distribution of FGFR3 Alterations in BLCA-TCGA Cohort

Whole-exome sequencing (WES) and gene fusion events data from BLCA-TCGA cohort were used to study the relationship between layer classifications and FGFR3 alterations. WES data were obtained from firebrowse.org. Only FGFR3 mutations included in the erdafitinib technical sheet (R248C, S249C, G370C, and Y373C) were included in the analysis. Regarding FGFR3 fusions, data were obtained from Fusion Gene Database (https://ccsm.uth.edu/FusionGDB/, accessed on 18 January 2025). Only data about FGFR3-TACC3 fusions is available for BLCA-TCGA cohort.

### 4.11. Statistical Analyses

Comparisons between quantitative variables were performed using non-parametric Mann–Whitney and Kruskal–Wallis tests. *p*-values were adjusted using a Benjamini–Hochberg method. Comparisons of distributions were performed using a Chi-squared test, applying Fisher’s test when it was necessary. Survival analyses were performed using Kaplan–Meier and Cox regression. Disease-free survival (DFS) was calculated as the difference between date of relapse or last follow-up and date of TURBT. Statistical analyses were performed using Graph Pad Prism version 6.

## 5. Conclusions

To summarize, in this study we used high-throughput proteomics to characterize biological mechanisms involved in response and resistance to NACT in MIBC, employing both TURBT and cystectomy samples. Two independent classifications in TURBT samples were established. The first identified three proteomics groups, with one enriched for non-responder patients. This classification was not related to classical luminal and basal subtypes. Remarkably, this group of non-responder patients to NACT has higher protein expression of NECTIN4 and ERBB2, both relevant actionable targets in MIBC. The other classification identified two groups a priori not related with response to NACT that matches with luminal and basal groups. Interestingly, having a CR is a prognostic factor only in luminal tumors. Finally, cystectomies from non-responder patients were analyzed searching for mechanisms of resistance. The possible mechanisms of resistance identified in each group may be used as guidance for future therapeutic options.

## Figures and Tables

**Table 1:** Clinical characteristics of EPIC-MIBC cohort.

EPIC-MIBC Patients	n = 58
Gender	
Female	17 (29%)
ECOG	
0	45 (78%)
1	4 (7%)
Unknown	9 (15%)
Other histology than urothelial	
Papillary	13 (22%)
Squamous	2 (4%)
Papillary + squamous	3 (5%)
Unknown	40 (69%)
ypTNM satge	
0	18 (31%)
2	10 (17%)
3	13 (23%)
4	4 (7%)
Tis	10 (17%)
Unknown	3 (5%)
Prior treatment with BCG	
No	55 (95%)
Yes	3 (5%)
Chemotherapy scheme	
ddMVAC	25 (43%)
Cisplatin-gemcitabine	32 (55%)
Other	1 (2%)
Response to NACT	
Yes	24 (41%)
No	32 (55%)

## Data Availability

Proteomics raw data are available in the ProteomeXchange Consortium via the PRIDE (http://www.ebi.ac.uk/pride) partner repository with the dataset identifier PXD057995.

## Supplementary Materials

The following supporting information can be downloaded at https://www.mdpi.com/article/10.3390/ijms27010476/s1.

## Abbreviations

BIC	Bayesian Information Criterion
CC	Consensus clustering
CMS	Consensus Molecular Subtypes
CR	Complete response
DFS	Disease-free survival
DIA	Data-independent acquisition
FFPE	Formalin-fixed paraffin-embedded
GSEA	Gene Set Enrichment Analysis
NACT	Neoadjuvant chemotherapy
MIBC	Muscle-invasive bladder carcinoma
OS	Overall survival
PGM	Probabilistic Graphical Model
TCGA	The Cancer Genome Atlas
TURBT	Transurethral resection of bladder tumor
WES	Whole-exome sequencing

## References

[1] Bray F., Laversanne M., Sung H., Ferlay J., Siegel R.L., Soerjomataram I., Jemal A. (2024). Global cancer statistics 2022: GLOBOCAN estimates of incidence and mortality worldwide for 36 cancers in 185 countries. CA Cancer J. Clin..

[2] Alfred Witjes J., Max Bruins H., Carrión A., Cathomas R., Compérat E., Efstathiou J.A., Fietkau R., Gakis G., Lorch A., Martini A. (2024). European Association of Urology Guidelines on Muscle-invasive and Metastatic Bladder Cancer: Summary of the 2023 Guidelines. Eur. Urol..

[3] Bajorin D.F., Witjes J.A., Gschwend J.E., Schenker M., Valderrama B.P., Tomita Y., Bamias A., Lebret T., Shariat S.F., Park S.H. (2021). Adjuvant Nivolumab versus Placebo in Muscle-Invasive Urothelial Carcinoma. N. Engl. J. Med..

[4] Powles T., Catto J.W.F., Galsky M.D., Al-Ahmadie H., Meeks J.J., Nishiyama H., Vu T.Q., Antonuzzo L., Wiechno P., Atduev V. (2024). Perioperative Durvalumab with Neoadjuvant Chemotherapy in Operable Bladder Cancer. N. Engl. J. Med..

[5] Choi W., Porten S., Kim S., Willis D., Plimack E.R., Hoffman-Censits J., Roth B., Cheng T., Tran M., Lee I.L. (2014). Identification of distinct basal and luminal subtypes of muscle-invasive bladder cancer with different sensitivities to frontline chemotherapy. Cancer Cell.

[6] Robertson A.G., Kim J., Al-Ahmadie H., Bellmunt J., Guo G., Cherniack A.D., Hinoue T., Laird P.W., Hoadley K.A., Akbani R. (2017). Comprehensive Molecular Characterization of Muscle-Invasive Bladder Cancer. Cell.

[7] Trilla-Fuertes L., Gámez-Pozo A., Prado-Vázquez G., Zapater-Moros A., Díaz-Almirón M., Arevalillo J.M., Ferrer-Gómez M., Navarro H., Maín P., Espinosa E. (2019). Biological molecular layer classification of muscle-invasive bladder cancer opens new treatment opportunities. BMC Cancer.

[8] Kamoun A., de Reyniès A., Allory Y., Sjödahl G., Robertson A.G., Seiler R., Hoadley K.A., Groeneveld C.S., Al-Ahmadie H., Choi W. (2020). A Consensus Molecular Classification of Muscle-invasive Bladder Cancer. Eur. Urol..

[9] Necchi A., Raggi D., Gallina A., Ross J.S., Farè E., Giannatempo P., Marandino L., Colecchia M., Lucianò R., Bianchi M. (2020). Impact of Molecular Subtyping and Immune Infiltration on Pathological Response and Outcome Following Neoadjuvant Pembrolizumab in Muscle-invasive Bladder Cancer. Eur. Urol..

[10] Seiler R., Ashab H.A.D., Erho N., van Rhijn B.W.G., Winters B., Douglas J., Van Kessel K.E., Fransen van de Putte E.E., Sommerlad M., Wang N.Q. (2017). Impact of Molecular Subtypes in Muscle-invasive Bladder Cancer on Predicting Response and Survival after Neoadjuvant Chemotherapy. Eur. Urol..

[11] de Velasco G., Trilla-Fuertes L., Gamez-Pozo A., Urbanowicz M., Ruiz-Ares G., Sepúlveda J.M., Prado-Vazquez G., Arevalillo J.M., Zapater-Moros A., Navarro H. (2017). Urothelial cancer proteomics provides both prognostic and functional information. Sci. Rep..

[12] Groeneveld C.S., Sanchez-Quiles V., Dufour F., Shi M., Dingli F., Nicolle R., Chapeaublanc E., Poullet P., Jeffery D., Krucker C. (2024). Proteogenomic Characterization of Bladder Cancer Reveals Sensitivity to Apoptosis Induced by Tumor Necrosis Factor-related Apoptosis-inducing Ligand in FGFR3-mutated Tumors. Eur. Urol..

[13] Dressler F.F., Diedrichs F., Sabtan D., Hinrichs S., Krisp C., Gemoll T., Hennig M., Mackedanz P., Schlotfeldt M., Voß H. (2024). Proteomic analysis of the urothelial cancer landscape. Nat. Commun..

[14] López-Camacho E., Prado-Vázquez G., Martínez-Pérez D., Ferrer-Gómez M., Llorente-Armijo S., López-Vacas R., Díaz-Almirón M., Gámez-Pozo A., Vara J.F., Feliu J. (2023). A Novel Molecular Analysis Approach in Colorectal Cancer Suggests New Treatment Opportunities. Cancers.

[15] Prado-Vázquez G., Gámez-Pozo A., Trilla-Fuertes L., Arevalillo J.M., Zapater-Moros A., Ferrer-Gómez M., Díaz-Almirón M., López-Vacas R., Navarro H., Maín P. (2019). A novel approach to triple-negative breast cancer molecular classification reveals a luminal immune-positive subgroup with good prognoses. Sci. Rep..

[16] Robertson J.F., Lindemann J.P., Llombart-Cussac A., Rolski J., Feltl D., Dewar J., Emerson L., Dean A., Ellis M.J. (2012). Fulvestrant 500 mg versus anastrozole 1 mg for the first-line treatment of advanced breast cancer: Follow-up analysis from the randomized ‘FIRST’ study. Breast Cancer Res. Treat..

[17] Petrella G., Ciufolini G., Vago R., Cicero D.O. (2020). The Interplay between Oxidative Phosphorylation and Glycolysis as a Potential Marker of Bladder Cancer Progression. Int. J. Mol. Sci..

[18] Mosaad Y.M. (2015). Clinical Role of Human Leukocyte Antigen in Health and Disease. Scand. J. Immunol..

[19] Piao X.M., Kang H.W., Jeong P., Byun Y.J., Lee H.Y., Kim K., Seo S.P., Kim W.T., Lee J.Y., Ha Y.S. (2021). A prognostic immune predictor, HLA-DRA, plays diverse roles in non-muscle invasive and muscle invasive bladder cancer. Urol. Oncol..

[20] Kim Y., Ju H., Yoo S.Y., Jeong J., Heo J., Lee S., Park J.M., Yoon S.Y., Jeong S.U., Lee J. (2023). Glutathione dynamics is a potential predictive and therapeutic trait for neoadjuvant chemotherapy response in bladder cancer. Cell Rep. Med..

[21] Klümper N., Brägelmann J., Bahlinger V., Hartmann A., Grünwald V., Kuppe C., Hölzel M., Eckstein M. (2025). Membranous expression of target protein is required for ADC response in urothelial cancer. Eur. Urol..

[22] Brave M.H., Maguire W.F., Weinstock C., Zhang H., Gao X., Li F., Yu J., Fu W., Zhao H., Pierce W.F. (2024). FDA Approval Summary: Enfortumab Vedotin plus Pembrolizumab for Locally Advanced or Metastatic Urothelial Carcinoma. Clin. Cancer Res..

[23] Powles T., Valderrama B.P., Gupta S., Bedke J., Kikuchi E., Hoffman-Censits J., Iyer G., Vulsteke C., Park S.H., Shin S.J. (2024). Enfortumab Vedotin and Pembrolizumab in Untreated Advanced Urothelial Cancer. N. Engl. J. Med..

[24] Chu C.E., Sjöström M., Egusa E.A., Gibb E.A., Badura M.L., Zhu J., Koshkin V.S., Stohr B.A., Meng M.V., Pruthi R.S. (2021). Heterogeneity in *NECTIN4* Expression Across Molecular Subtypes of Urothelial Cancer Mediates Sensitivity to Enfortumab Vedotin. Clin. Cancer Res..

[25] Ogitani Y., Aida T., Hagihara K., Yamaguchi J., Ishii C., Harada N., Soma M., Okamoto H., Oitate M., Arakawa S. (2016). DS-8201a, A Novel HER2-Targeting ADC with a Novel DNA Topoisomerase I Inhibitor, Demonstrates a Promising Antitumor Efficacy with Differentiation from T-DM1. Clin. Cancer Res..

[26] Meric-Bernstam F., Makker V., Oaknin A., Oh D.Y., Banerjee S., González-Martín A., Jung K.H., Ługowska I., Manso L., Manzano A. (2024). Efficacy and Safety of Trastuzumab Deruxtecan in Patients With HER2-Expressing Solid Tumors: Primary Results From the DESTINY-PanTumor02 Phase II Trial. J. Clin. Oncol..

[27] Sonpavde G., Goldman B.H., Speights V.O., Lerner S.P., Wood D.P., Vogelzang N.J., Trump D.L., Natale R.B., Grossman H.B., Crawford E.D. (2009). Quality of pathologic response and surgery correlate with survival for patients with completely resected bladder cancer after neoadjuvant chemotherapy. Cancer.

[28] Perera T.P.S., Jovcheva E., Mevellec L., Vialard J., De Lange D., Verhulst T., Paulussen C., Van De Ven K., King P., Freyne E. (2017). Discovery and Pharmacological Characterization of JNJ-42756493 (Erdafitinib), a Functionally Selective Small-Molecule FGFR Family Inhibitor. Mol. Cancer Ther..

[29] Li R., Linscott J., Catto J.W.F., Daneshmand S., Faltas B.M., Kamat A.M., Meeks J.J., Necchi A., Pradere B., Ross J.S. (2025). FGFR Inhibition in Urothelial Carcinoma. Eur. Urol..

[30] Plimack E.R., Dunbrack R.L., Brennan T.A., Andrake M.D., Zhou Y., Serebriiskii I.G., Slifker M., Alpaugh K., Dulaimi E., Palma N. (2015). Defects in DNA Repair Genes Predict Response to Neoadjuvant Cisplatin-based Chemotherapy in Muscle-invasive Bladder Cancer. Eur. Urol..

[31] Fan B., Zheng X., Wang Y., Hu X. (2023). Predicting prognosis and clinical efficacy of immune checkpoint blockade therapy. Pathol. Oncol. Res..

[32] Olkhov-Mitsel E., Hodgson A., Liu S.K., Vesprini D., Bayani J., Bartlett J.M.S., Xu B., Downes M.R. (2022). Upregulation of IFNγ-mediated chemokines dominate the immune transcriptome of muscle-invasive urothelial carcinoma. Sci. Rep..

[33] Fus Ł., Górnicka B. (2016). Role of angiogenesis in urothelial bladder carcinoma. Cent. Eur. J. Urol..

[34] Elayat G., Punev I., Selim A. (2023). An Overview of Angiogenesis in Bladder Cancer. Curr. Oncol. Rep..

[35] Narayanan S., Srinivas S. (2017). Incorporating VEGF-targeted therapy in advanced urothelial cancer. Ther. Adv. Med. Oncol..

[36] Gámez-Pozo A., Berges-Soria J., Arevalillo J.M., Nanni P., López-Vacas R., Navarro H., Grossmann J., Castaneda C., Main P., Díaz-Almirón M. (2015). Combined label-free quantitative proteomics and microRNA expression analysis of breast cancer unravel molecular differences with clinical implications. Cancer Res..

[37] Gámez-Pozo A., Ferrer N.I., Ciruelos E., López-Vacas R., Martínez F.G., Espinosa E., Vara J. (2013). Shotgun proteomics of archival triple-negative breast cancer samples. Proteom. Clin. Appl..

[38] Türker C., Akal F., Joho D., Panse C., Barkow-Oesterreicher S., Rehrauer H., Schlapbach R. (2010). B-Fabric: The Swiss Army Knife for Life Sciences. Proceedings of the 13th International Conference on Extending Database Technology (EDBT ‘10).

[39] Demichev V., Messner C.B., Vernardis S.I., Lilley K.S., Ralser M. (2020). DIA-NN: Neural networks and interference correction enable deep proteome coverage in high throughput. Nat. Methods.

[40] Tyanova S., Temu T., Sinitcyn P., Carlson A., Hein M.Y., Geiger T., Mann M., Cox J. (2016). The Perseus computational platform for comprehensive analysis of (prote)omics data. Nat. Methods.

[41] Ritchie M., Phipson B., Wu D., Hu Y., Law C., Shi W., Smyth G. (2015). *limma* powers differential expression analyses for RNA-sequencing and microarray studies. Nucleic Acid Res..

[42] Witten D.M., Tibshirani R. (2010). A framework for feature selection in clustering. J. Am. Stat. Assoc..

[43] Wilkerson M.D., Hayes D.N. (2010). ConsensusClusterPlus: A class discovery tool with confidence assessments and item tracking. Bioinformatics.

[44] Huang D.W., Sherman B.T., Lempicki R.A. (2009). Systematic and integrative analysis of large gene lists using DAVID bioinformatics resources. Nat. Protoc..

[45] Lauritzen S. (1996). Graphical Models.

[46] Abreu G., Edwards D., Labouriau R. (2010). High-Dimensional Graphical Model Search with the gRapHD R Package. J. Stat. Softw..

[47] Blondel V.D., Guillaume J.-L., Lambiotte R., Lefebvre E. (2008). Fast unfolding of communities in large networks. J. Stat. Mech. Theory Exp..

[48] Subramanian A., Tamayo P., Mootha V.K., Mukherjee S., Ebert B.L., Gillette M.A., Paulovich A., Pomeroy S.L., Golub T.R., Lander E.S. (2005). Gene set enrichment analysis: A knowledge-based approach for interpreting genome-wide expression profiles. Proc. Natl. Acad. Sci. USA.

