# Supplementary material for: Identification of a Muscle-Invasive Bladder Carcinoma Molecular Subtype of Poor Responders to Neoadjuvant Chemotherapy and High Expression of Targetable Biomarkers
Source: Int J Mol Sci. 2026 Jan 2;27(1):476. doi: 10.3390/ijms27010476 (PMC12787041; doi:10.3390/ijms27010476)
Supplement: Supplementary file 1 [file ijms-27-00476-s001.zip › Sup figures.pdf]

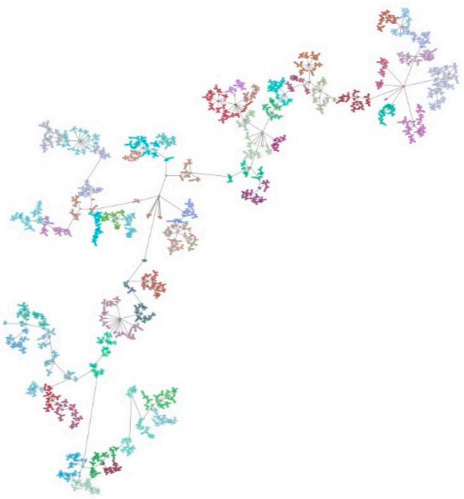

Sup Fig 1: Protein network of transurethral resection samples from EPIC muscle-invasive bladder carcinoma patients built using probabilistic graphical models. Each color represents a functional node defined by Louvain community algorithm. Functions of each node determined by gene ontology analyses can be found at Sup Table 1.

mRNA processsing & spliceosome

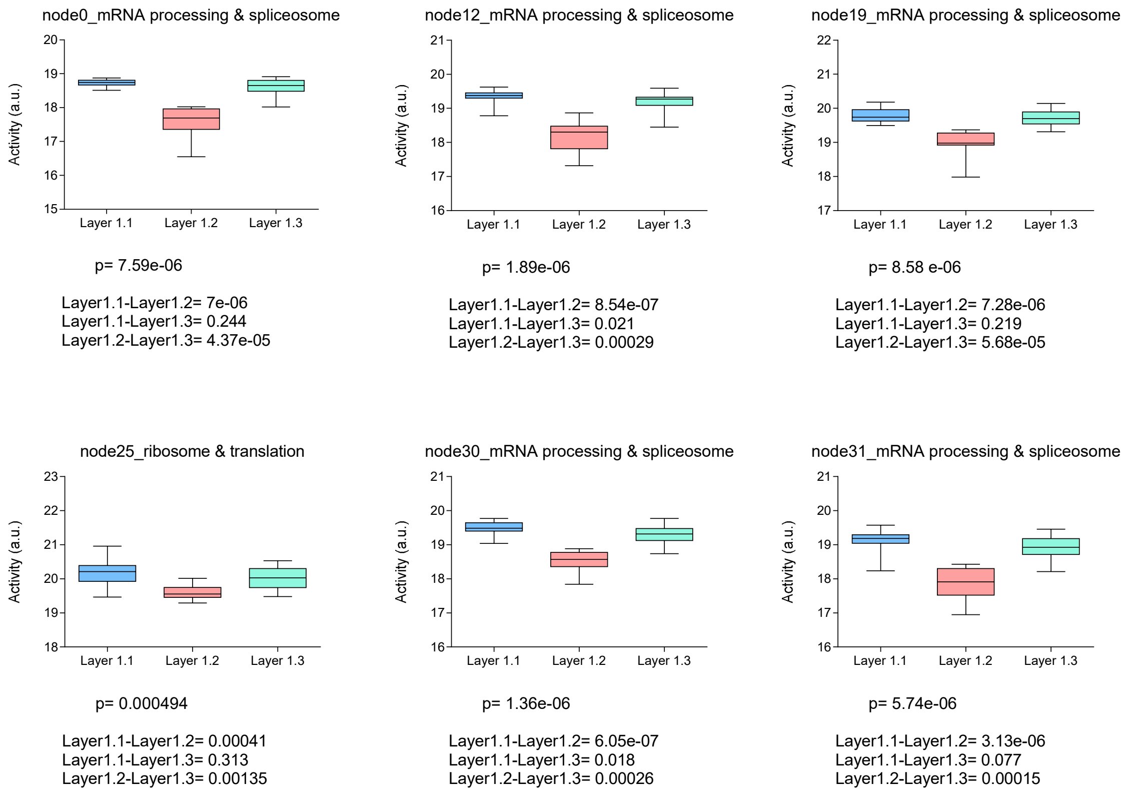

node32_mRNA processing & spliceosome

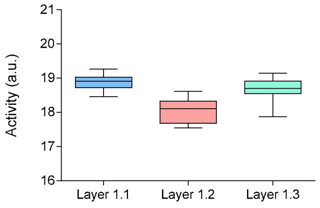

p= 1.5e-05

Layer1.1-Layer1.2= 7.88e-06
Layer1.1-Layer1.3= 0.069
Layer1.2-Layer1.3= 0.00040

node38_RNA Processing

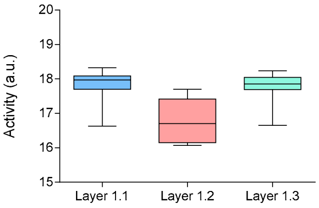

p= 6.96e-05

Layer1.1-Layer1.2= 8.66e-05
Layer1.1-Layer1.3= 0.436
Layer1.2-Layer1.3= 0.00014

node39_mRNA transport

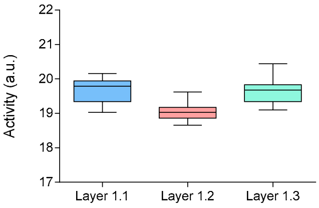

p= 0.00014

Layer1.1-Layer1.2= 0.00015
Layer1.1-Layer1.3= 0.393
Layer1.2-Layer1.3= 0.00033

node42_RNA transport

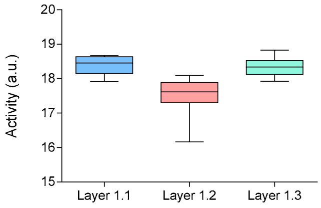

p= 2.61e-05

Layer1.1-Layer1.2= 3.37e-05
Layer1.1-Layer1.3= 0.413
Layer1.2-Layer1.3= 6.35e-05

node45_mRNA processing & spliceosome

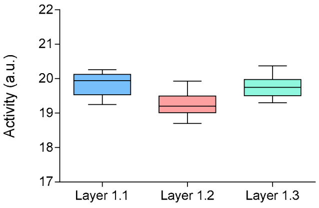

p= 0.00092

Layer1.1-Layer1.2= 0.00071
Layer1.1-Layer1.3= 0.278
Layer1.2-Layer1.3= 0.00278

node47_endoplasmic reticulum transport

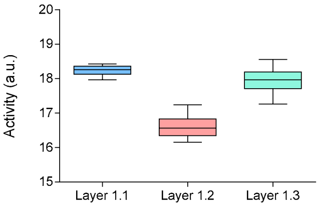

p= 1.03e-07

Layer1.1-Layer1.2= 5.07e-08
Layer1.1-Layer1.3= 0.0017
Layer1.2-Layer1.3= 0.00041

Proliferation

node1_regulation of nucleotide-excision repair, regulation of cell cycle

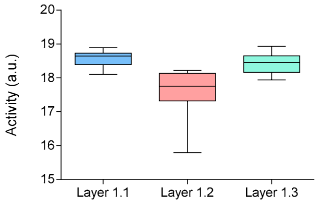

p= 2.42e-05

Layer1.1-Layer1.2= 1.26e-05
Layer1.1-Layer1.3= 0.063
Layer1.2-Layer1.3= 0.00067

node13_cell-cell adhesion, cell migration

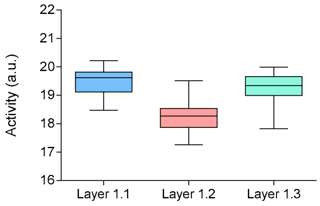

p= 0.00014

Layer1.1-Layer1.2= 0.0001
Layer1.1-Layer1.3= 0.203
Layer1.2-Layer1.3= 0.00078

node17_DNA repair

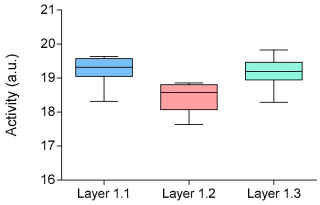

p= 0.000123

Layer1.1-Layer1.2= 0.000132
Layer1.1-Layer1.3= 0.39
Layer1.2-Layer1.3= 0.00028

node18_DNA replication

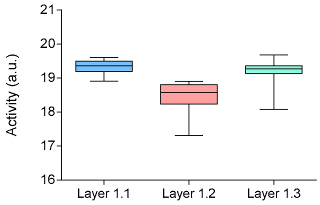

p= 1.21e-05

Layer1.1-Layer1.2= 7.88e-06
Layer1.1-Layer1.3= 0.137
Layer1.2-Layer1.3= 0.000143

node52_telomeres, microtubules

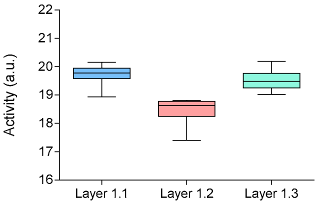

p= 1.37e-06

Layer1.1-Layer1.2= 6.32e-07
Layer1.1-Layer1.3= 0.032
Layer1.2-Layer1.3= 0.00013

Immune response

node4_adaptive immune response, antigen binding, immunoglobulin

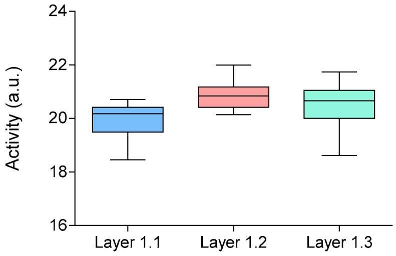

p= 0.0029

Layer1.1-Layer1.2= 0.0073
Layer1.1-Layer1.3= 0.0077
Layer1.2-Layer1.3= 0.281

node5_Immune response, defense response

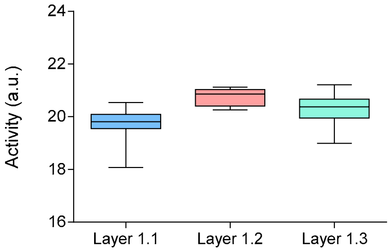

p= 8.67e-05

Layer1.1-Layer1.2= 9.29e-05
Layer1.1-Layer1.3= 0.0048
Layer1.2-Layer1.3= 0.028

node6_Immune response

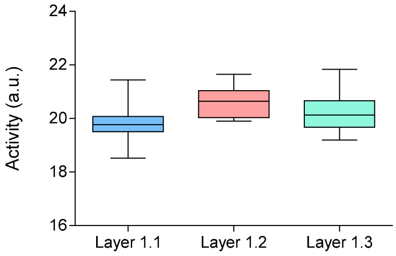

p= 0.025

Layer1.1-Layer1.2= 0.024
Layer1.1-Layer1.3= 0.127
Layer1.2-Layer1.3= 0.131

node7_Immune response, defense response

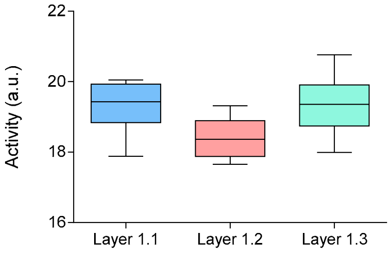

p= 0.0065

Layer1.1-Layer1.2= 0.005
Layer1.1-Layer1.3= 0.863
Layer1.2-Layer1.3= 0.005

Metabolism

node14_metabolic process

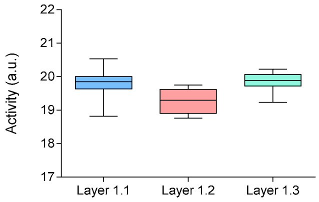

p= 0.00081

Layer1.1-Layer1.2= 0.0045
Layer1.1-Layer1.3= 0.479
Layer1.2-Layer1.3= 0.00052

node16_cellular detoxification

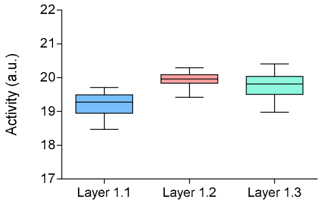

p= 7.56e-06

Layer1.1-Layer1.2= 5.35e-05
Layer1.1-Layer1.3= 5.35e-05
Layer1.2-Layer1.3= 0.23

node46_mitochondrial metabolism

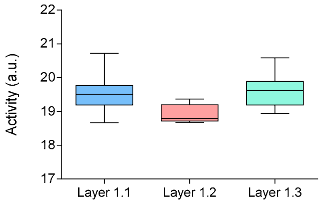

p= 0.00046

Layer1.1-Layer1.2= 0.00442
Layer1.1-Layer1.3= 0.369
Layer1.2-Layer1.3= 0.000278

Ribosome &translation

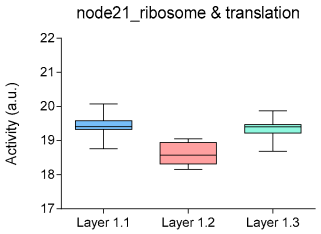

p= 2.76 e-05

Layer1.1-Layer1.2= 3.91e-05
Layer1.1-Layer1.3= 0.454
Layer1.2-Layer1.3= 5.28e-05

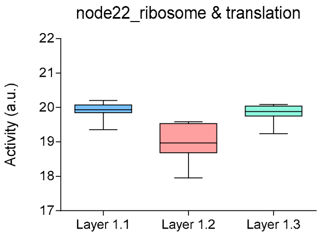

p= 2.76 e-05

Layer1.1-Layer1.2= 2.7e-05
Layer1.1-Layer1.3= 0.314
Layer1.2-Layer1.3= 9.54e-05

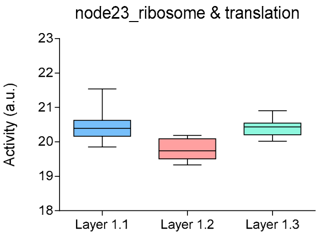

p= 0.000148

Layer1.1-Layer1.2= 0.00034
Layer1.1-Layer1.3= 0.919
Layer1.2-Layer1.3= 0.000153

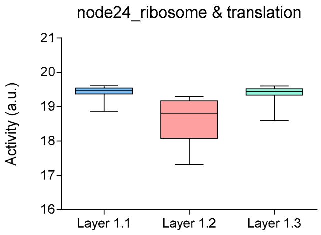

p= 0.000102

Layer1.1-Layer1.2= 0.00012
Layer1.1-Layer1.3= 0.452
Layer1.2-Layer1.3= 0.000196

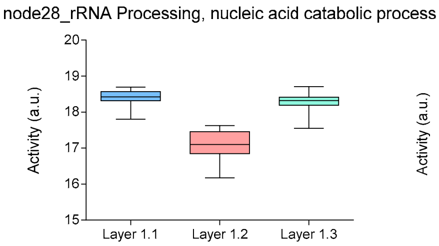

p= 2.77e-06

Layer1.1-Layer1.2= 1.42e-06
Layer1.1-Layer1.3= 0.058
Layer1.2-Layer1.3= 0.00017

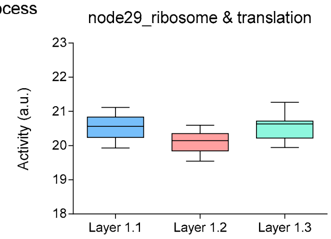

p= 0.0098

Layer1.1-Layer1.2= 0.0094
Layer1.1-Layer1.3= 0.944
Layer1.2-Layer1.3= 0.0094

Others

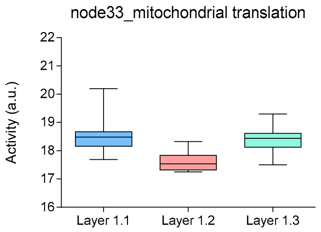

p= 0.00013

Layer1.1-Layer1.2= 0.00022
Layer1.1-Layer1.3= 0.933
Layer1.2-Layer1.3= 0.00017

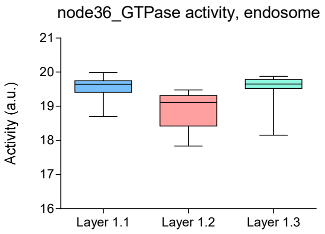

p= 0.00038

Layer1.1-Layer1.2= 0.00095
Layer1.1-Layer1.3= 0.823
Layer1.2-Layer1.3= 0.00034

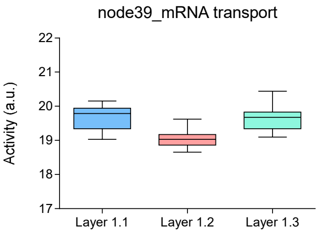

p= 0.00014

Layer1.1-Layer1.2= 0.00015
Layer1.1-Layer1.3= 0.393
Layer1.2-Layer1.3= 0.00033

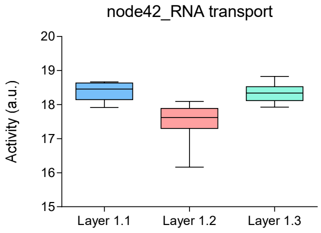

p= 2.61e-05

Layer1.1-Layer1.2= 3.37e-05
Layer1.1-Layer1.3= 0.413
Layer1.2-Layer1.3= 6.35e-05

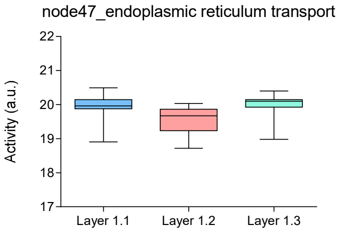

p= 0.0018

Layer1.1-Layer1.2= 0.00115
Layer1.1-Layer1.3= 0.39
Layer1.2-Layer1.3= 0.00116

Sup Fig 2: Differential functional node activities in EPIC-MIBC TURBT samples according to Layer1 groups.

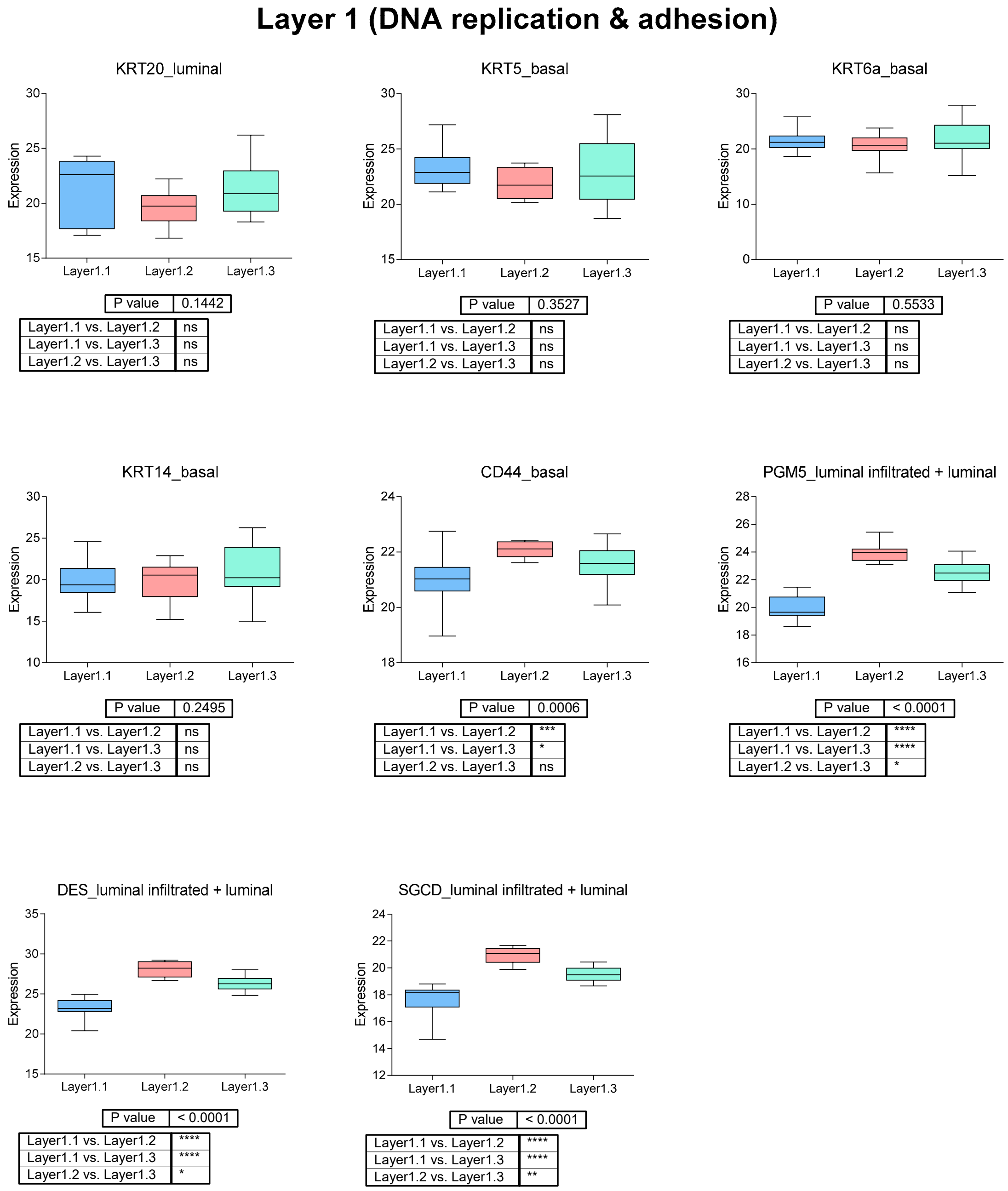

Sup Fig 3: Expression of luminal and basal biomarkers in Layer1 groups in EPIC-MIBC TURBT samples. ns p> 0.05; * p ≤ 0.05; ** p ≤ 0.01; *** p ≤ 0.001; **** p ≤ 0.0001.

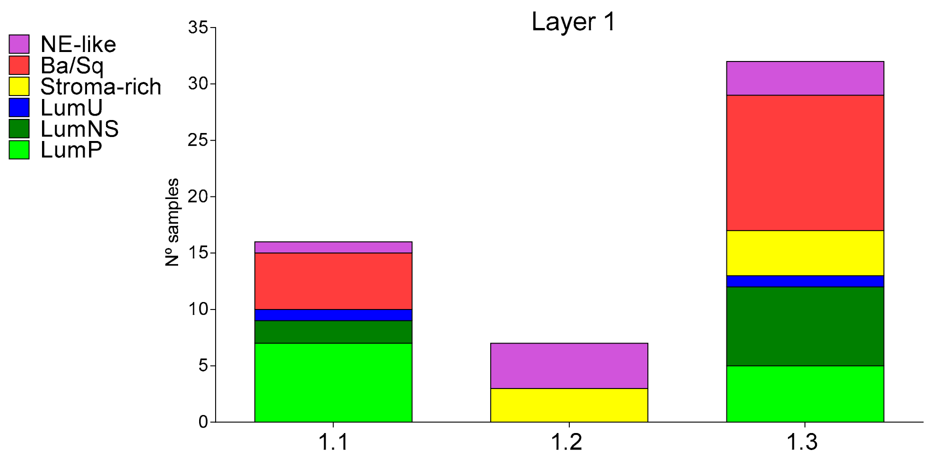

Sup Fig 4: Distribution of MIBC consensus molecular subtypes in Layer1 groups of EPIC-MIBC TURBT samples.

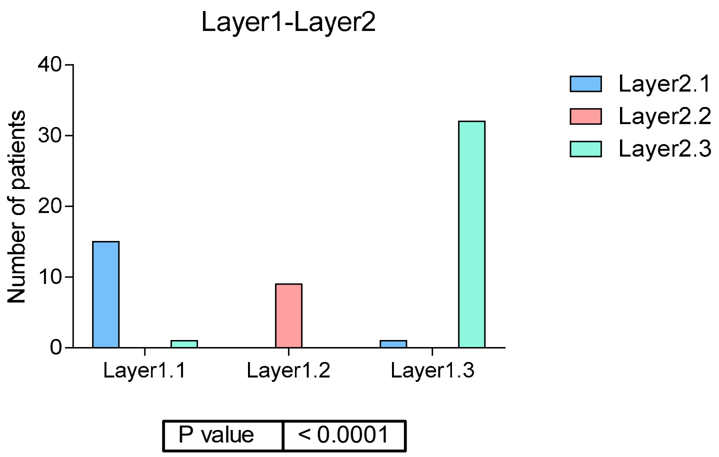

Sup Fig 5: Distribution of Layer1 and Layer2 classifications.

mRNA processing

node0_mRNA processing & spliceosome

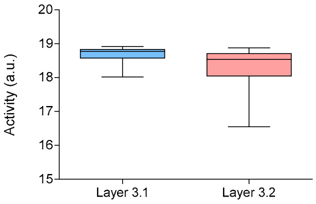

p=0.0013

node9_mRNA processing & spliceosome

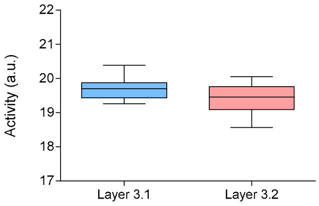

p=0.015

node25_mRNA processing & spliceosome

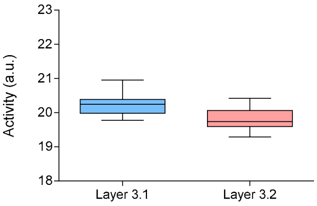

p=0.0

node30_mRNA processing & spliceosome

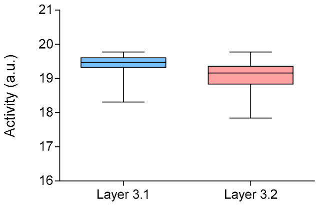

p=0.0015

node31_mRNA processing

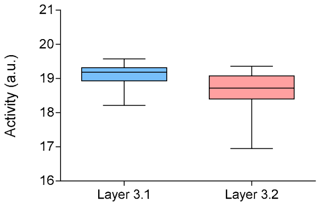

p=0.00019

node32_mRNA processing & spliceosome

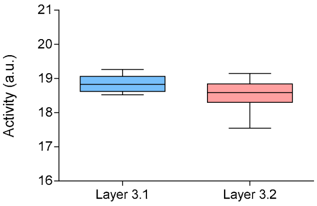

p=0.0016

node45_mRNA processing & spliceosome

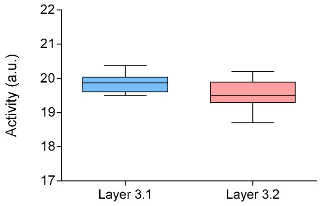

p=0.0033

node48_mRNA Processing

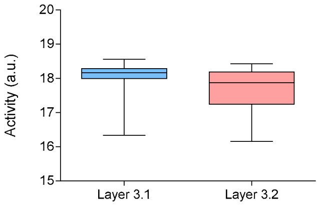

p=0.0169

Cytoskeleton

node3_cytoskeleton

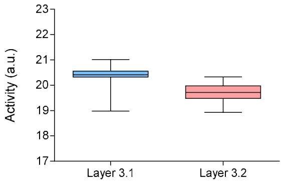

p=0.0047

node10_cytoskeleton, actin

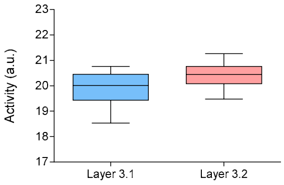

p=0.005

node11_focal adhesion & cytoskeleton

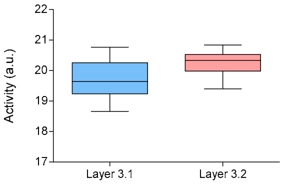

p=0.0002

node27_extracellular matrix, collagen

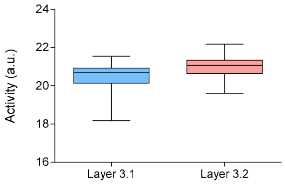

p=0.008

node40_extracellular matrix

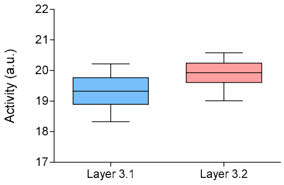

p=0.00016

node41_cytoskeleton, actin

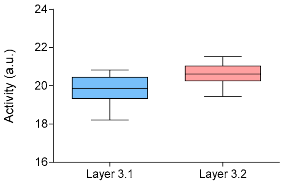

p=0.00017

node50_extracellular matrix

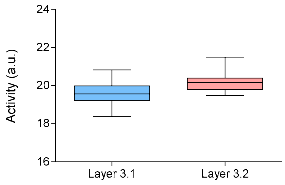

p=0.000468

node51_regulation of actin filament organization

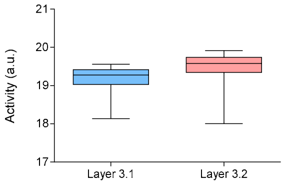

p=0.00012

node52_telomeres, microtubules

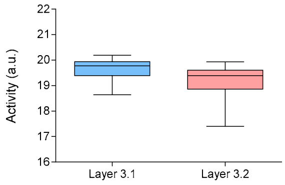

p=0.00124

Immune response

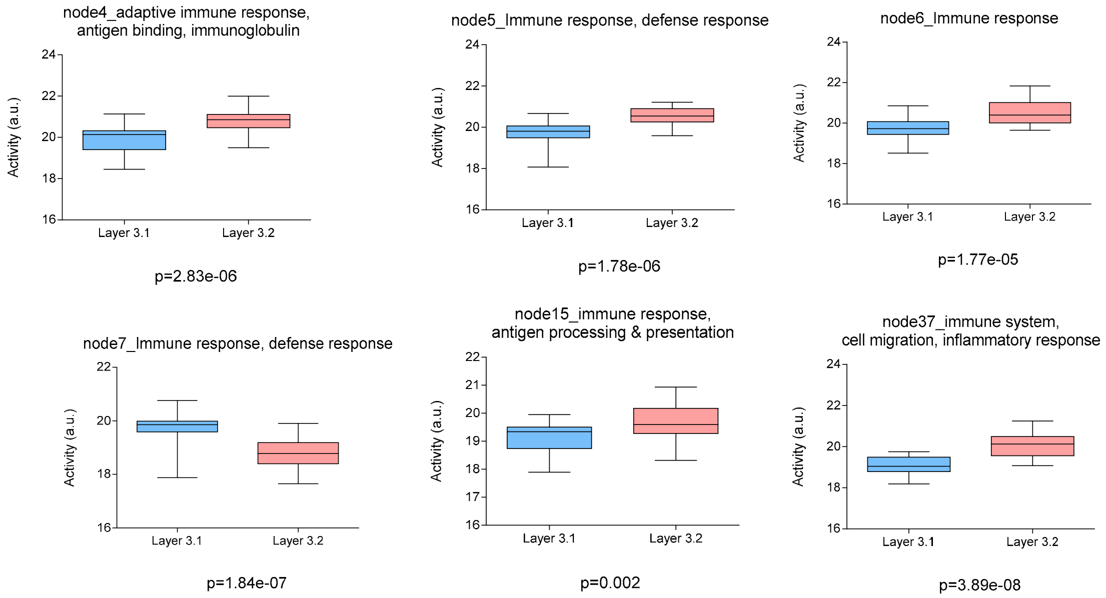

Mitochondria

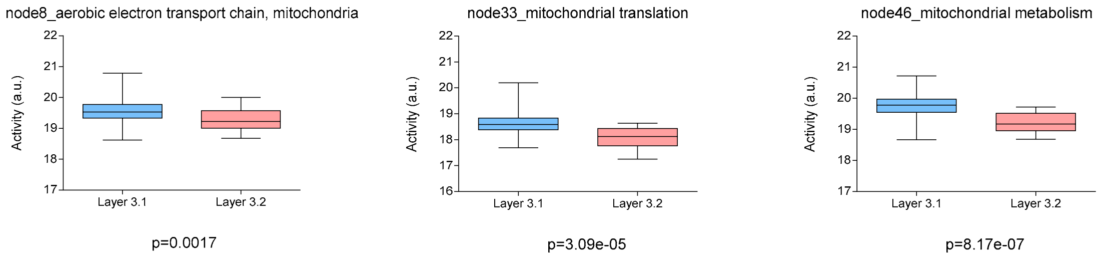

Proliferation

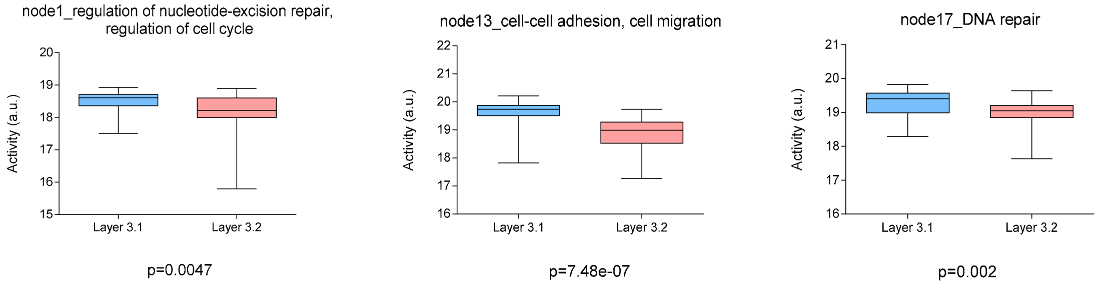

Transport

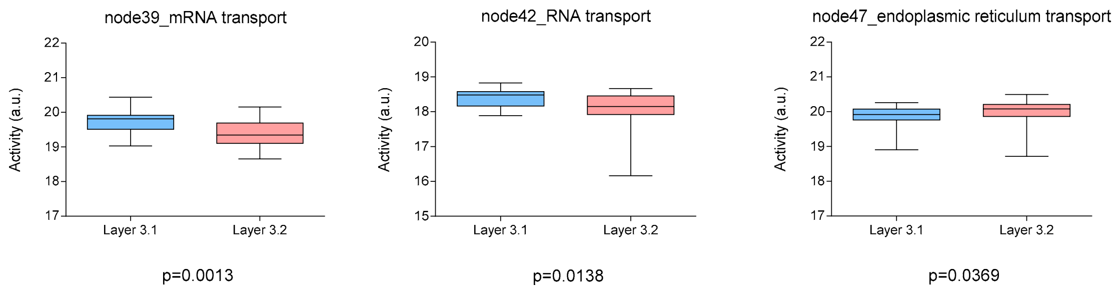

Sup Fig 6: Differential functional node activities in EPIC-MIBC TURBT samples according to Layer3 groups.

Layer 3-immune

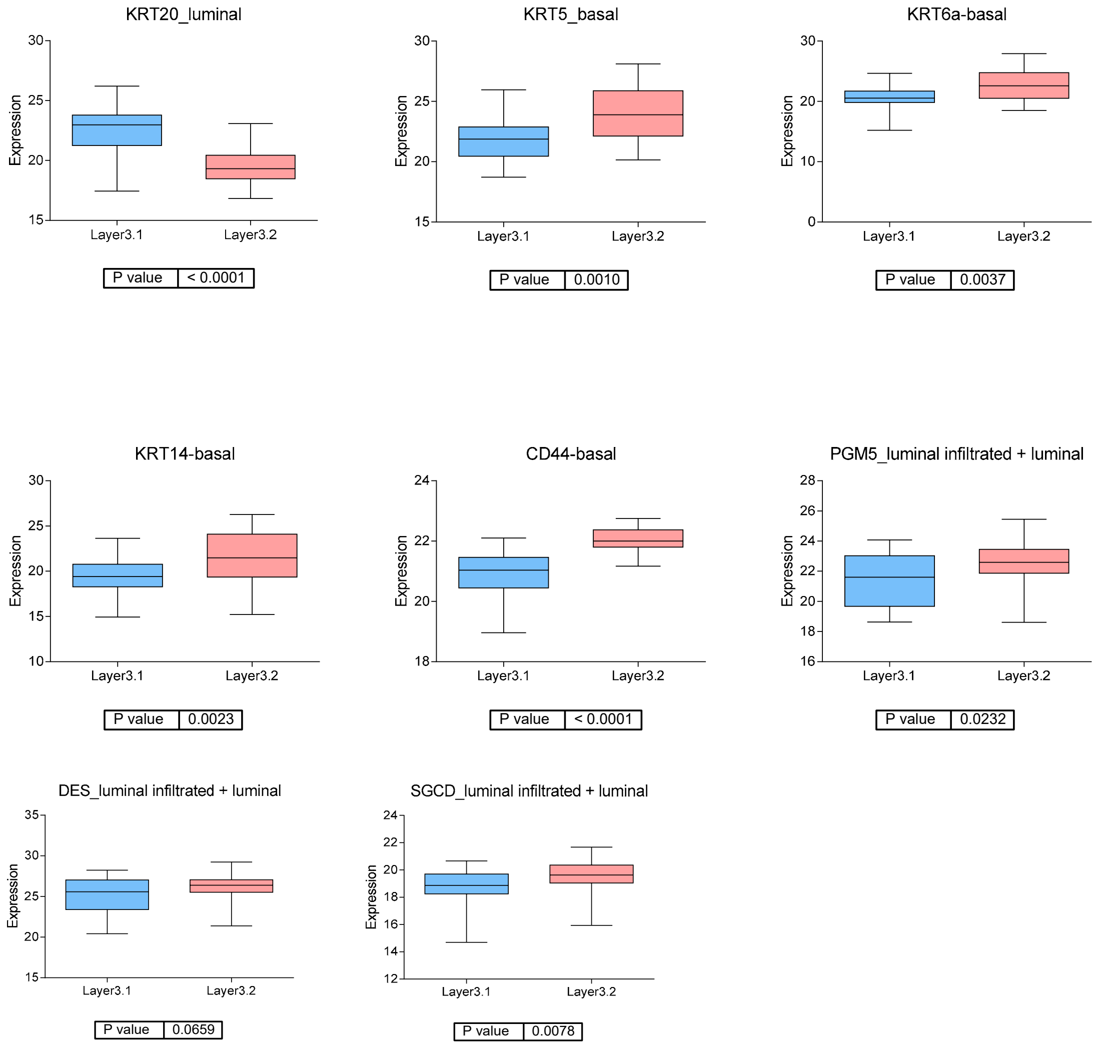

Sup Figure 7: Expression of luminal and basal biomarkers in Layer3 groups in EPIC-MIBC TURBT samples.

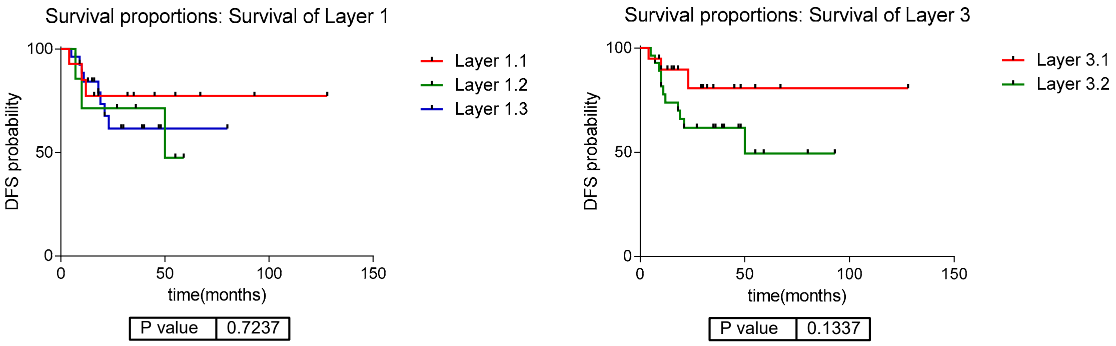

Hazard Ratio (logrank)	A/B	B/A
Ratio (and its reciprocal)	0.3923	2.549
95% CI of ratio	0.1518 to 1.277	0.7829 to 6.588

Sup Fig 8: Disease-free survival in each Layer classification.

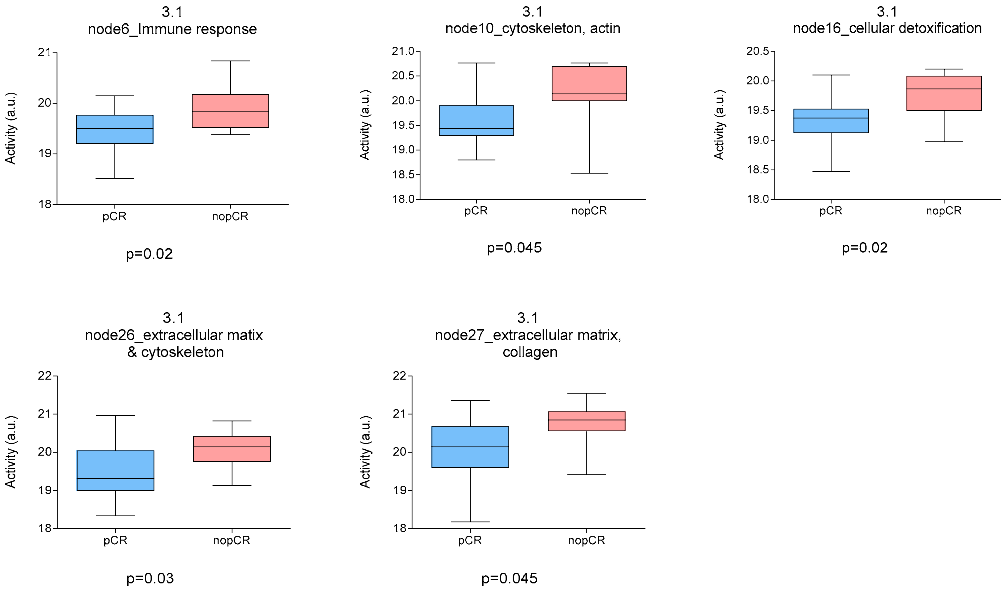

Sup Fig 9: Differential functional node activities according to response to NACT in samples from Layer3.1 group, which includes Luminal tumors.

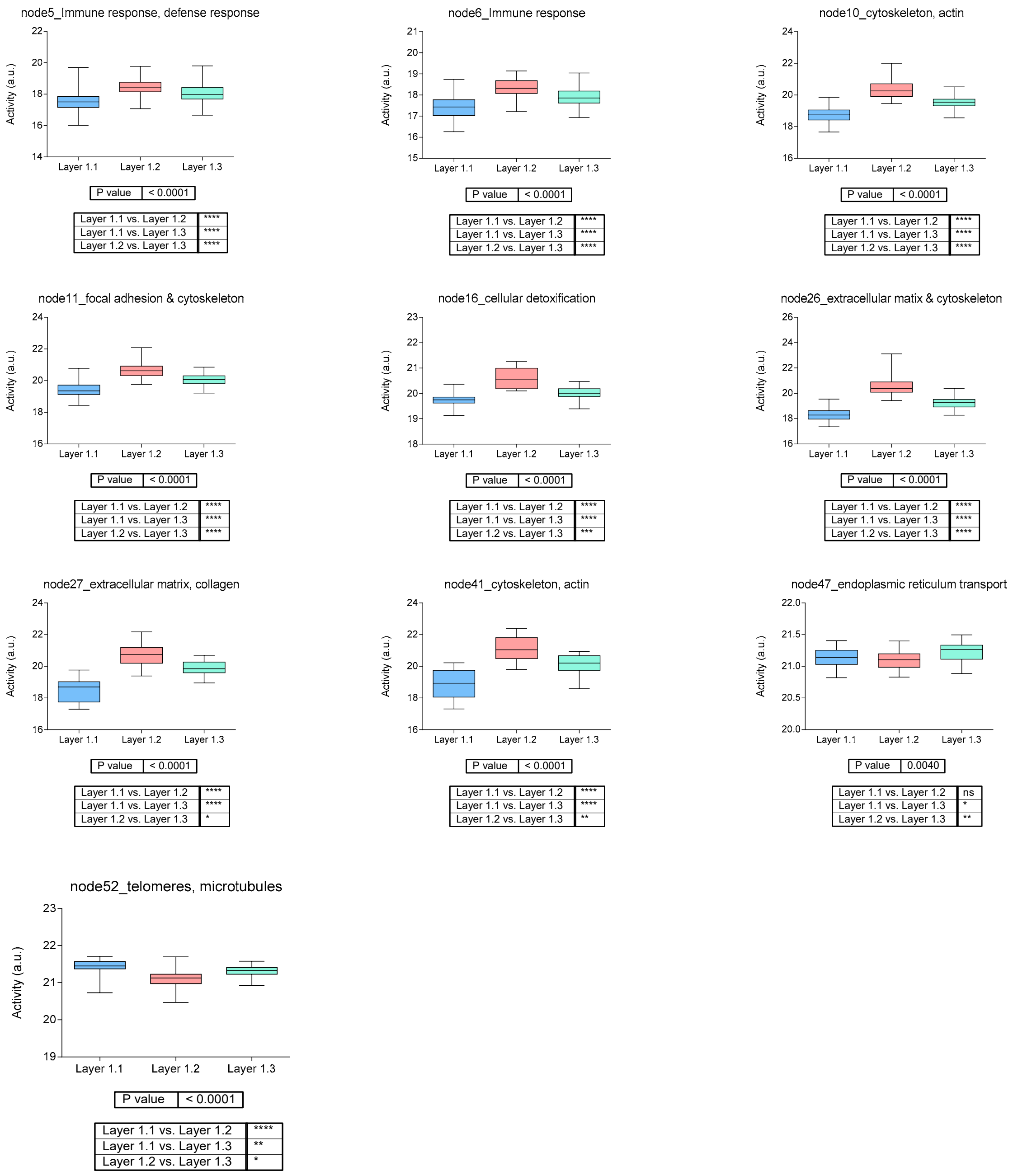

Sup Fig 10: Differential functional node activities in BLA-TCGA cohort according to Layer1 groups.

being more frequent in Layer1.1 and Layer3.1 (Fig 6).

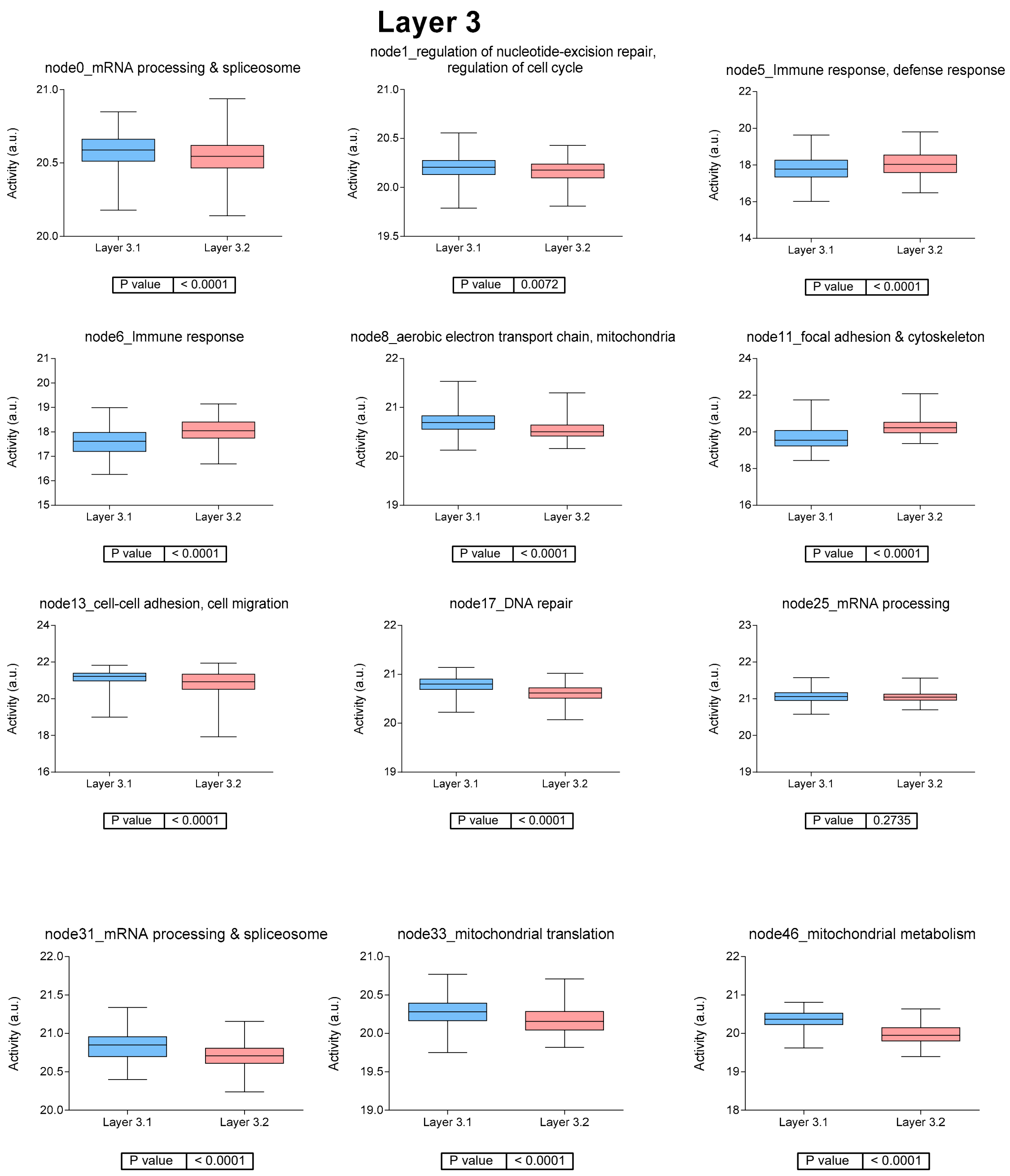

Sup Fig 11: Differential functional node activities in BLA-TCGA cohort according to Layer3 groups.
