# Supplementary material for: Identification of a Muscle-Invasive Bladder Carcinoma Molecular Subtype of Poor Responders to Neoadjuvant Chemotherapy and High Expression of Targetable Biomarkers
Source: Int J Mol Sci. 2026 Jan 2;27(1):476. doi: 10.3390/ijms27010476 (PMC12787041; doi:10.3390/ijms27010476)
Supplement: Supplementary file 1 [file ijms-27-00476-s001.zip › Sup tables.pdf]

Sup Table 1: Proteins that composes each functional node of the protein network defined using proteomics data in TURBT samples from the EPIC MIBC cohort.

Sup Table 2: List of proteins that were used to classify MIBC samples into Layer 1 groups.

Sup Table 3: List of proteins that were used to classify MIBC samples into Layer 3 groups.
